# Supplementary material for: Topological invariant and anomalous edge modes of strongly nonlinear systems
Source: Nat Commun. 2022 Jun 13;13:3379. doi: 10.1038/s41467-022-31084-y (PMC9192757; doi:10.1038/s41467-022-31084-y)
Supplement: Supplementary file 1 — Supplementary Information [file 41467_2022_31084_MOESM1_ESM.pdf]

Topological Invariant and Anomalous Edge States of Strongly Nonlinear Systems: Supplementary Information

Di Zhou,[1,*] D. Zeb Rocklin,[2] Michael Leamy,[2] and Yugui Yao[1,†]

[1]*Key Lab of Advanced Optoelectronic Quantum Architecture and Measurement (MOE) and School of Physics, Beijing Institute of Technology, Beijing 100081, China*

[2]*School of Physics, Georgia Institute of Technology, Atlanta, GA 30332, USA*

I. BERRY PHASE OF NONLINEAR BULK MODES

In this section, we derive Berry phase of nonlinear bulk modes by adiabatically evolving the wave function as the wave number q slowly traverses the Brillouin zone. We consider the nonlinear problem described by a classical two-field generalized nonlinear Schrödinger equations presented in the main text,

$$i\partial_t\Psi_n^{(1)} = \epsilon_0\Psi_n^{(1)} + f_1(\Psi_n^{(1)},\Psi_n^{(2)}) + f_2(\Psi_n^{(1)},\Psi_{n-1}^{(2)}),$$
$$i\partial_t\Psi_n^{(2)} = \epsilon_0\Psi_n^{(2)} + f_1(\Psi_n^{(2)},\Psi_n^{(1)}) + f_2(\Psi_n^{(2)},\Psi_{n+1}^{(1)}), \quad (1.1)$$

where $f_i(x,y)$ for $i=1,2$ are real-coefficient general polynomials of x, x^*, y, and y^*. Berry phase is derived from this general model.

In the linear limit, the interactions are approximated as $f_i(x,y)\approx c_i y$ ($c_{i=1,2}>0$). The model is a 2×2 matrix problem in which the bands are gapped. As the amplitude rises, nonlinearities become increasingly significant and the linear bulk modes evolve into nonlinear bulk modes. In this section, we study the simple case that the nonlinear bulk modes are non-degenerate and are stable. In other words, a nonlinear bulk mode is unique, provided that the amplitude A, the frequency ω, and the wave number q are given. In addition to these properties, we consider the simple case that the nonlinear bandgap [1, 2] never closes. As such, this system is a nonlinear extension of the linear two-band model.

We begin by defining the nonlinear periodic bulk mode of the system as follows,

$$\Psi_n(t) = \Psi_q(\omega t - qn) = \begin{pmatrix} \Psi_q^{(1)}(\omega t - qn) \\ \Psi_q^{(2)}(\omega t - qn + \phi_q) \end{pmatrix}, \quad (1.2)$$

where q is the wave number and ω is the frequency that belongs to the upper band of the nonlinear band structure. As such, the wave functions depend on the single variable $\theta=\omega t - qn$. This form is widely used in existing literatures to describe plane waves of strongly nonlinear normal modes [3]. For example, Jacobi-Elliptic plane waves are studied in nonlinear gravitational waves for classical Minkowskian Yang-Mills theory [4], where q identifies the site-to-site phase shift and the wave shape of the gravitational wave. Nonlinear plane waves arise in a number of other nonlinear classical systems as well, such as compressible atmosphere [5], porous media [6], and mechanical lattices [7, 8]. Their wave phase shift and waveforms are also controlled by the wavenumber q.

This ansatz always correctly states the periodicity of *nonlinear plane waves* based on the following three reasons. First, typical studies of weakly nonlinear bulk modes [7–13] via the method of multiple-scale reveal that all Fourier components are captured by the single θ variable, $\Psi_n=\sum_l \psi_{l,n}e^{il\theta}$. Second, numerical experiments such as shooting method (see Figs.1(b), 11(a,c), and Refs. [14–16]) manifests non-dispersive bulk modes in strongly nonlinear regime, which appear to be plane-wave like modes. Finally, analytic solutions for special wave numbers $q=0,\pi$ demonstrate that strongly nonlinear bulk modes are in line with Eq.(1.2). It is at this point that we adopt Eq.(1.2) as the general form of nonlinear bulk modes.

However, the ansatz in Eq.(1.2) is *not universal* to state the periodicity of all nonlinear modes. In particular, the ansatz fails for soliton modes and nonlinear localized modes. Dark and bright soliton modes manifest minimum and maximum amplitudes at the centers of the excitations, respectively. Eq.(1.2) demands that the amplitudes of the nonlinear mode are spatially uniform for all unit cells, which clearly violates the features of solitons. Soliton excitations cannot exist in systems subjected to periodic boundary conditions [7, 15], whereas the ansatz in Eq.(1.2) still holds for periodically arranged lattices. Nonlinear localized modes are a special class of excitations that cannot exist in purely linear systems. These modes stem from the bifurcation of nonlinearity. In contrast to soliton modes, nonlinear localized modes can emerge in lattices under periodic boundary conditions. The ansatz in Eq.(1.2) cannot cover these unconventional excitations because nonlinear localized modes are spatially localized at certain sites of the system and are clearly not plane-wave like states. In conclusion, although the ansatz in Eq.(1.2) is universal to state the periodicity of *nonlinear plane waves*, it is not capable of covering non-plane-wave modes like soliton excitations and nonlinear localized modes. These non-plane-wave states are unique for nonlinear systems, and they cannot be written in the format of Eq.(1.2).

In general, the wave forms of $\Psi_q^{(j)}(\theta)$ for $j=1,2$ are not sinusoidal in θ. We note that because the wave function component $\Psi_q^{(j)}(\theta)$ is 2π-periodic, it is defined up to

* dizhou@bit.edu.cn
† ygyao@bit.edu.cn

an arbitrary phase condition. In this paper, the phase condition is chosen by asking that when $\theta = 0$, the real part of wave component $\mathrm{Re}\,\Psi_q^{(j)}(\theta)$ reaches its amplitude/maximum,

$$\mathrm{Re}\,\Psi_q^{(j)}(\theta = 0) = \max(\mathrm{Re}\,\Psi_q^{(j)}(\theta)) \overset{\text{def}}{=} A, \quad j = 1, 2. \tag{1.3}$$

Note that the phase condition in Eq.(1.3) is similar to that in exponential functions, where $\mathrm{Re}\,e^{i\theta=0} = \max(\mathrm{Re}\,e^{i\theta})$. Following this convention, ϕ_q in Eq.(1.2) characterizes the relative phase between $\Psi_q^{(1)}$ and $\Psi_q^{(2)}$. The nonlinear mode has to fulfill the differential equation parametrized by wave number q,

$$i\partial_t \Psi_q(\theta) = H(\Psi_q), \tag{1.4}$$

where $\theta = \omega t$, and the nonlinear function $H(\Psi_q)$ is given by

$$H(\Psi_q) = \begin{pmatrix} \epsilon_0 \Psi_q^{(1)}(\theta) \\ \epsilon_0 \Psi_q^{(2)}(\theta + \phi_q) \end{pmatrix} + \begin{pmatrix} f_1(\Psi_q^{(1)}(\theta), \Psi_q^{(2)}(\theta + \phi_q)) \\ f_1(\Psi_q^{(2)}(\theta + \phi_q), \Psi_q^{(1)}(\theta)) \end{pmatrix} + \begin{pmatrix} f_2(\Psi_q^{(1)}(\theta), \Psi_q^{(2)}(\theta + q + \phi_q)) \\ f_2(\Psi_q^{(2)}(\theta + \phi_q), \Psi_q^{(1)}(\theta - q)) \end{pmatrix}. \tag{1.5}$$

In what follows, we study the nonlinear bulk mode with the fixed amplitude A. Therefore, the mode frequency ω, the relative phase ϕ_q, and the waveform are controlled by the wave number q.

Next, we adiabatically evolve the wave number $q(t)$ traversing the Brillouin zone from $q(0) = q$ to $q(t) = q + 2\pi$. According to the nonlinear extension of adiabatic theorem [17–20], a system initially in one of its nonlinear mode Ψ_q of the upper band will stay as an instantaneous upper-band nonlinear mode of $H(\Psi_{q(t)})$ throughout the process. This theorem is valid when the control parameter q varies sufficiently slowly compared to the frequencies [18], and the nonlinear bulk modes are stable [19] within the amplitude scope of this paper. In Sec.II.E and Sec.III, we exploit analytic Floquet analysis and numerical self-oscillation methods [8] to doubly confirm the stability of these nonlinear modes. Hence the only degree of freedom is the phase of the mode. At time t, the mode is

$$\Psi_{q(t)}\left(\int_0^t \omega(t', q(t'))dt' - \gamma(t)\right). \tag{1.6}$$

We are interested in the extra phase term γ, which will be carried out as follows. Substituting Eq.(1.6) into Eq.(1.4), we have

$$\frac{d\gamma}{dt}\frac{\partial \Psi_q}{\partial \theta} = \frac{dq}{dt}\frac{\partial \Psi_q}{\partial q}, \tag{1.7}$$

where $\theta = \omega t$ stands for the phase of the wave function Ψ_q. We bare in mind that the nonlinear bulk mode is 2π-periodic in its phase, which grants Fourier transformation. We expand $\Psi_q^{(j)}(\theta)$, the component of periodic wave function, in terms of its Fourier series:

$$\Psi_q^{(j)}(\theta) = \sum_{l \in \mathcal{Z}} \psi_{l,q}^{(j)} e^{-il\theta} \qquad j = 1, 2, \tag{1.8}$$

where $\psi_{l,q}^{(j)}$ is the l-th Fourier component of $\Psi_q^{(j)}$ (l is integer). Inserting Eq.(1.8) into Eq.(1.7), we have

$$\frac{d\gamma}{dt}\sum_l ile^{-il\theta}\begin{pmatrix} \psi_{l,q}^{(1)} \\ \psi_{l,q}^{(2)} e^{-il\phi_q} \end{pmatrix} = -\frac{dq}{dt}\sum_l e^{-il\theta}\begin{pmatrix} \partial \psi_{l,q}^{(1)}/\partial q \\ e^{-il\phi_q}[\partial \psi_{l,q}^{(2)}/\partial q - il\psi_{l,q}^{(2)}(\partial \phi_q/\partial q)] \end{pmatrix}. \tag{1.9}$$

We multiply Eq.(1.9) on both sides by $\Psi_q^\dagger(\theta)$ and integrate θ from 0 to 2π, to obtain the following result,

$$\frac{d\gamma}{dt}\sum_{l'} il'\left(|\psi_{l',q}^{(1)}|^2 + |\psi_{l',q}^{(2)}|^2\right) = \frac{dq}{dt}\sum_l \left(il|\psi_{l,q}^{(2)}|^2\frac{\partial \phi_q}{\partial q} - \psi_{l,q}^{(1)*}\frac{\partial \psi_{l,q}^{(1)}}{\partial q} - \psi_{l,q}^{(2)*}\frac{\partial \psi_{l,q}^{(2)}}{\partial q}\right), \tag{1.10}$$

where the integration $\int_0^{2\pi} d\theta\, e^{i(l'-l)\theta} = 2\pi\delta_{ll'}$ has been used. Since the wave number q traverses the Brillouin zone, by integrating over time t we obtain the phase term γ expressed in terms of a loop integration through the entire Brillouin zone,

$$\gamma = \oint_{\mathrm{BZ}} A_q dq, \tag{1.11}$$

where

$$A_q = \frac{\sum_l \left(l|\psi_{l,q}^{(2)}|^2\frac{\partial \phi_q}{\partial q} + i\sum_j \psi_{l,q}^{(j)*}\frac{\partial \psi_{l,q}^{(j)}}{\partial q}\right)}{\sum_{l'} l'\left(\sum_{j'} |\psi_{l',q}^{(j')}|^2\right)} \tag{1.12}$$

is Berry connection of general-nonlinear systems. Eq.(1.11) is Berry phase of the upper-band nonlinear bulk modes, which is the generalization of Berry phase in linear and Kerr-nonlinear problems.

Recent progress [21] reveals Kerr-nonlinear topological phases and chiral surface currents in a (2+1)-dimensional photonic metamaterial, where the topological number is the 3-form Chern-Simons theory. Our quantized Berry phase is the 1-form Chern-Simons theory, which characterizes topological charge of 1-dimensional systems. Despite the distinct physical and mathematical origins, the topological invariants of Ref. [21] and our work are both constructed from Berry connection. Ref. [21] constructs Berry connection under Kerr-nonlinear interactions, whereas our formalism establishes this notion for general nonlinearities. Substituting Kerr-nonlinearity into Eq.(3) of the main text arrives at the Kerr-nonlinear Berry connection that works for Ref. [21] (details in Sec.VI). Thus, the general-nonlinear Berry connection

of our work covers the Kerr-nonlinear one, and finds the Kerr-nonlinearity-induced topological transitions in Ref. [21].

Having established Berry phase of nonlinear bulk modes, we now build the connection between Eq.(1.11) and its conventional form in linear systems. In quantum mechanics, Schrödinger equation $i\partial_t\Psi(t) = H\Psi(t)$ is linear in $\Psi(t)$, where H is the Hamiltonian as a linear operator, ω are the eigenvalues, and the eigenmodes $\Psi(t) = \Psi e^{-i\omega t}$ are sinusoidal in time. Let us consider a 1D lattice of diatomic unit cells subjected to periodic boundary condition. Translational symmetry allows plane-wave eigenmodes $\Psi(t) = \Psi_q e^{iqn-i\omega t} = (\Psi_q^{(1)}, \Psi_q^{(2)} e^{-i\phi_q})^\top e^{iqn-i\omega t}$, where q is the wave number, ϕ_q is the relative phase between the two parts of the wave function, and $\sum_{j=1,2}|\Psi_q^{(j)}|^2 \equiv 1$ is the normalization condition. The phase condition is chosen such that both $\Psi_q^{(j=1,2)}$ are real, which is consistent with Eq.(1.3). Thus, $\Psi_q^{(j)}(\theta) = \Psi_q^{(j)} e^{-i\theta}$, where $\theta = \omega t - qn$. According to Eq.(1.8), the Fourier components are that $\psi_{l,q}^{(j)} = \Psi_q^{(j)}\delta_{l,1}$, which greatly simplify Eq.(1.11) to the following form,

$$\begin{aligned}
\gamma &= \oint_{\mathrm{BZ}} dq \frac{\sum_l \left(l|\psi_{l,q}^{(2)}|^2 \frac{\partial\phi_q}{\partial q} + i\sum_j \psi_{l,q}^{(j)*}\frac{\partial\psi_{l,q}^{(j)}}{\partial q}\right)\delta_{l1}}{\sum_{l'} l' \left(\sum_{j'}|\psi_{l',q}^{(j')}|^2\right)\delta_{l'1}} \\
&= \oint_{\mathrm{BZ}} dq \left(|\psi_{1,q}^{(2)}|^2 \frac{\partial\phi_q}{\partial q} + i\sum_j \psi_{1,q}^{(j)*}\frac{\partial\psi_{1,q}^{(j)}}{\partial q}\right) \\
&= \oint_{\mathrm{BZ}} dq\, i \left(\sum_j \Psi_q^{(j)*}\partial_q\Psi_q^{(j)} - i|\Psi_q^{(2)}|^2\partial_q\phi_q \right) \\
&= \oint_{\mathrm{BZ}} dq\, i(\Psi_q^{(1)*}, \Psi_q^{(2)*}e^{i\phi_q})\partial_q \begin{pmatrix} \Psi_q^{(1)} \\ \Psi_q^{(2)}e^{-i\phi_q} \end{pmatrix} \\
&= \oint_{\mathrm{BZ}} dq\, i\langle\Psi_q|\partial_q|\Psi_q\rangle = \gamma_{\mathrm{linear}},
\end{aligned} \quad (1.13)$$

where $\Psi_q = (\Psi_q^{(1)}, \Psi_q^{(2)}e^{-i\phi_q})^\top$ is the eigenvector of the Hamiltonian, and γ_{linear} denotes the conventional form of Berry phase in linear systems.

Next, we briefly review a reflection-symmetric linear model and quantized γ_{linear}, where the equations of motion

$$\begin{aligned}
i\partial_t\Psi_n^{(1)} &= \epsilon_0\Psi_n^{(1)} + c_1\Psi_n^{(2)} + c_2\Psi_{n-1}^{(2)}, \\
i\partial_t\Psi_n^{(2)} &= \epsilon_0\Psi_n^{(2)} + c_1\Psi_n^{(1)} + c_2\Psi_{n+1}^{(1)}
\end{aligned} \quad (1.14)$$

are subjected to periodic boundary condition, $\epsilon_0 > 0$ is the on-site potential, and $c_i > 0$ for $i = 1$ and $i = 2$ stand for intra-cell and inter-cell couplings, respectively. In wavevector space, the motion equations are reduced to $i\partial_t\Psi_q = H_q\Psi_q$, where $H_q = \epsilon_0 I_2 + (c_1 + c_2\cos q)\sigma_x + (c_2\sin q)\sigma_y$. The eigenvalue of the upper band reads $\omega = \epsilon_0 + |c_1 + c_2e^{iq}|$, and the associated eigenvector is $\Psi_q = (1, (c_1 + c_2e^{iq})/|c_1 + c_2e^{iq}|)^\top/\sqrt{2}$. We invoke Eq.(1.13) to reduce Berry phase to the form, $\gamma_{\mathrm{linear}} = \frac{i}{2}\oint_{\mathrm{BZ}} dq\left[\partial_q \ln(c_1 + c_2e^{iq}) - \partial_q \ln|c_1 + c_2e^{iq}|\right]$, which can be interpreted in two ways. In the first way, we notice that the second part vanishes, because the length of $|c_1 + c_2e^{iq}|$ does not wind around the origin when integrated over the Brillouin zone. The second way is to denote $c_1 + c_2e^{iq} = \rho_q e^{-i\phi_q}$, and then Berry phase simply represents how ϕ_q winds around the origin by 0 or 2π when q traverses the Brillouin zone. Thus, γ_{linear} can be reduced to

$$\gamma_{\mathrm{linear}} = \frac{i}{2}\oint_{\mathrm{BZ}} dq\,\partial_q \ln(c_1 + c_2e^{iq}) = \frac{1}{2}\oint_{\mathrm{BZ}} dq\,\partial_q\phi_q. \quad (1.15)$$

In Ref. [22], the topological index is captured by the winding number $\mathcal{N} = -(2\pi i)^{-1}\oint_{\mathrm{BZ}} dq\,\partial_q \ln\det C(q) = (2\pi)^{-1}\oint_{\mathrm{BZ}} dq\,\partial_q\phi_q$, where $C(q) = c_1 + c_2e^{iq}$ is the compatibility matrix that describes floppy modes. Thus, Berry phase and winding number are related by $\gamma_{\mathrm{linear}} = \mathcal{N}/\pi$. In summary, Eq.(1.11) is the nonlinear extension of the topological index in Ref. [22].

II. SYMMETRIES OF GENERALIZED NONLINEAR SCHRÖDINGER EQUATIONS AND THE QUANTIZATION OF BERRY PHASE

In this section, we study symmetry properties of the model in Eqs.(1.1). We prove that the frequencies of nonlinear bulk modes are restricted to be real numbers due to the combined effect of time-reversal symmetry and spatial reflection symmetry. Then, we demonstrate that Berry phase in Eq.(1.11) is quantized by reflection symmetry.

A. Time-reversal symmetry

Here, we demonstrate that the model in Eqs.(1.1) is subjected to time-reversal symmetry, as long as the interactions yield the constraint

$$f_i^*(x, y) = f_i(x^*, y^*), \quad (2.1)$$

which is met by any real-coefficient polynomials of x, x^*, y, y^*, including the minimal model of the main text. The considered nonlinear solution $\Psi_n(t)$ satisfies the equations of motion $i\partial_t\Psi_n(t) = H(\Psi_n)$, where $H(\Psi_n)$ is the nonlinear function of $\Psi_n(t)$ elucidated by Eqs.(1.1). Taking complex conjugation on both sides offers us a new equation

$$i\partial_t\Psi_n^*(-t) = H^*(\Psi_n(-t)). \quad (2.2)$$

Substituting Eq.(2.1), we arrive at the new result,

$$i\partial_t\Psi_n^*(-t) = H(\Psi_n^*(-t)). \quad (2.3)$$

Eq.(2.3) suggests that given a nonlinear solution $\Psi_n(t)$, we can always find a partner solution $\Psi_n^*(-t)$ for the same equations of motion. Consequently, the model respects

time-reversal symmetry, in the sense that nonlinear solutions $\Psi_n(t)$ and $\Psi_n^*(-t)$ always come in pairs [23].

For given amplitude A, time-reversal symmetry demands that the frequencies of nonlinear bulk modes are related by $\omega(q) = \omega^*(-q)$. To prove this, we consider a nonlinear bulk mode

$$\Psi_q = (\Psi_q^{(1)}(\omega t - qn), \Psi_q^{(2)}(\omega t - qn + \phi_q))^\top, \quad (2.4)$$

where q is the wave number, and $\omega = \omega(q)$. Ψ_q is a solution of Eqs.(1.1) only if it fulfills Eq.(1.4), which is equivalent to the following nonlinear differential equation,

$$\Psi_q(\theta = \omega t - qn) = (\Psi_q^{(1)}(\theta), \Psi_q^{(2)}(\theta + \phi_q))^\top : \\ \mathcal{L}(\Psi_q) = 0, \quad (2.5)$$

where the nonlinear differential operator $\mathcal{L}(\Psi_q)$ is defined as follows,

$$\mathcal{L}(\Psi_q) = (i\omega\partial_\theta - \epsilon_0) \begin{pmatrix} \Psi_q^{(1)}(\theta) \\ \Psi_q^{(2)}(\theta) \end{pmatrix} - \begin{pmatrix} f_1(\Psi_q^{(1)}(\theta), \Psi_q^{(2)}(\theta + \phi_q)) \\ f_1(\Psi_q^{(2)}(\theta), \Psi_q^{(1)}(\theta - \phi_q)) \end{pmatrix} - \begin{pmatrix} f_2(\Psi_q^{(1)}(\theta), \Psi_q^{(2)}(\theta + q + \phi_q)) \\ f_2(\Psi_q^{(2)}(\theta), \Psi_q^{(1)}(\theta - q - \phi_q)) \end{pmatrix}. \quad (2.6)$$

In general, the waveform of Ψ_q is not sinusoidal, which is the natural result of nonlinearity. Time-reversal symmetry demands a partner solution

$$\Psi_q^*(-t) = (\Psi_q^{(1)*}(-\omega t - qn), \Psi_q^{(2)*}(-\omega t - qn + \phi_q))^\top, \quad (2.7)$$

where $\omega = \omega(q)$. This mode also renders the equations of motion to vanish,

$$\Psi_q^*(\theta = -\omega t - qn) = (\Psi_q^{(1)*}(\theta), \Psi_q^{(2)*}(\theta + \phi_q))^\top : \\ \mathcal{L}(\Psi_q^*(-t)) = [\mathcal{L}(\Psi_q)]^* = 0. \quad (2.8)$$

We note that the wave number and frequency of the mode are $-q$ and $\omega(-q)$, respectively. Hence, Eqs.(2.8) demonstrates the following relationship,

$$\omega(-q) = \omega^*(q). \quad (2.9)$$

In the following subsection, we will prove that together with reflection symmetry, the frequencies are constrained to be real numbers (i.e., $\omega^* = \omega$), and nonlinear bulk modes are periodic in time.

Time-reversal symmetry does not serve to quantize nonlinear Berry phase. It just helps to demonstrate the real-valuedness of nonlinear bulk mode frequencies. In fact, numerical shooting method and analytic method of multiple-scale already manifest real frequencies of nonlinear bulk modes. We use time-reversal symmetry to mathematically confirm this result.

B. Reflection symmetry and quantized Berry phase

Before going into details of reflection symmetry in the nonlinear system, we briefly review this symmetry in the linearized model and demonstrate the quantization of Berry phase, when the coupling is linearized as $f_i(x, y) = c_i y$. We convert the wave function into wavevector space $\Psi_n = \Psi_q e^{i(qn - \omega t)}$, to reduce the equations of motion as $H_q\Psi_q = \omega\Psi_q$, where $H_q = \epsilon_0 I + (c_1 + c_2\cos q)\sigma_x + (c_2 \sin q)\sigma_y$, and $\sigma_{x,y,z}$ are Pauli matrices. H_q is subjected to reflection symmetry, meaning that one can find a reflection symmetry operator $M_x = \sigma_x$, such that $M_x^2 = I$, and $M_x H_q M_x^{-1} = H_{-q}$. We notice $H_{-q}M_x\Psi_q = \omega M_x \Psi_q$. It demonstrates that Ψ_q and Ψ_{-q} are related by $M_x\Psi_q = e^{i\phi_q}\Psi_{-q}$, where ϕ_q is the phase factor connecting Ψ_q and Ψ_{-q}. At high-symmetry points when $q_{\text{hs}} = 0, \pi$ ("hs" is short for high symmetry), we find that M_x and H_q commute, which demands the phase factor $\phi_{\text{hs}} = 0$ or π. Finally, in the linear problem, we prove the quantization of Berry phase by showing that $\gamma = \phi_\pi - \phi_0 = 0$ or $\pi \mod 2\pi$.

We now proceed to investigate the nonlinear problem raised in Eqs.(1.1). We notice that the nonlinear system is subjected to reflection symmetry: the equations of motion are invariant under the reflection transformation,

$$(\Psi_n^{(1)}, \Psi_n^{(2)}) \to (\Psi_{-n}^{(2)}, \Psi_{-n}^{(1)}). \quad (2.10)$$

In Eq.(2.4), given a nonlinear bulk mode solution Ψ_q that renders $\mathcal{L}(\Psi_q)$ to vanish, Eq.(2.10) demands a new nonlinear bulk mode solution $\Psi'_{-q} = (\Psi_q^{(2)}(\omega t + qn), \Psi_q^{(1)}(\omega t + qn - \phi_q))^\top$ that also renders $\mathcal{L}(\Psi'_{-q})$ to vanish,

$$\Psi'_{-q}(\theta = \omega t + qn) = (\Psi_q^{(2)}(\theta), \Psi_q^{(1)}(\theta - \phi_q))^\top : \\ \mathcal{L}(\Psi'_{-q}) = \sigma_x\mathcal{L}(\Psi_q) = 0, \quad (2.11)$$

where $\omega = \omega(q)$. Since the wave number and frequency of Ψ'_{-q} are $-q$ and $\omega(-q)$, respectively, we reach the conclusion

$$\omega(-q) = \omega(q). \quad (2.12)$$

Together with Eq.(2.9), we show $\omega(q) = \omega^*(q)$ for all q, which means the frequencies of nonlinear bulk modes are real. From now on, we denote $\omega(q)$ as ω for simplicity, and Ψ'_{-q} is a nonlinear mode with frequency ω and wave number $-q$.

On the other hand, following the notation of Eq.(2.4), the nonlinear bulk mode of frequency ω and wave number $-q$ is by definition denoted as

$$\Psi_{-q}(\theta = \omega t + qn) = (\Psi_{-q}^{(1)}(\theta), \Psi_{-q}^{(2)}(\theta + \phi_{-q}))^\top. \quad (2.13)$$

Due to the non-degenerate nature of nonlinear bulk modes, Ψ_{-q} and Ψ'_{-q} have to be the same solution, which in turn imposes the constraints

$$\Psi_{-q}^{(1)}(\theta) = \Psi_q^{(2)}(\theta), \quad (2.14)$$

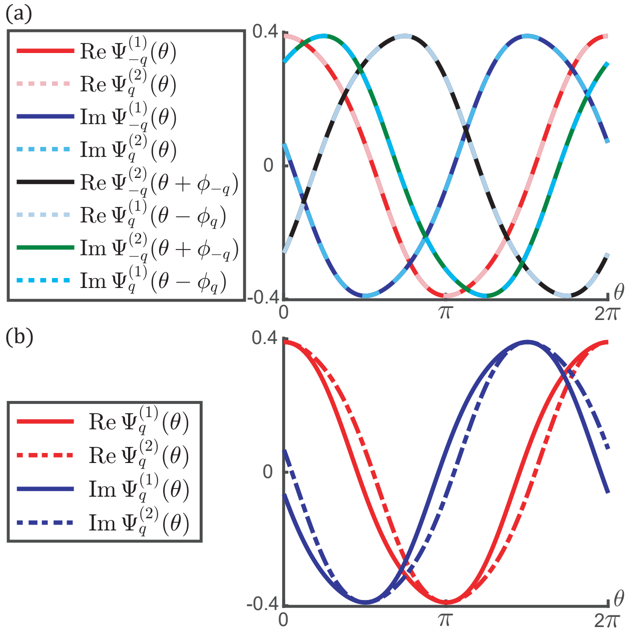

Supplementary Figure 1. Numerical verification of $\Psi^{(1)}_{-q}(\theta) = \Psi^{(2)}_{q}(\theta)$ and $-\phi_q = \phi_{-q}$ by computing nonlinear bulk modes. Here the nonlinear bulk modes are calculated in the lattice composed of classical dimer fields. The lattice is subjected to periodic boundary condition, and the interaction parameters are carried over from Fig.3 of the main text, where $\epsilon_0 = 0$, $c_1 = 0.25$, $c_2 = 0.37$, $d_1 = 0.22$, and $d_2 = 0.02$. (a) Numerical illustration of Eqs.(2.14, 2.15) by comparing nonlinear bulk modes $\Psi_{-q}(t)$ in Eq.(2.13) and $\Psi'_{-q}(t)$ in Eq.(2.11), where the wave number $q = 8\pi/9$ and the amplitude $A = 0.3873$. $\Psi^{(1)}_{-q}(\theta) = \Psi^{(2)}_{q}(\theta)$ and $-\phi_q = \phi_{-q}$ are verified by the perfect overlap between the wave functions. (b) Numerical demonstration of $\mathrm{Re}\,\Psi^{(1)}_{q}(\theta) \neq \mathrm{Re}\,\Psi^{(2)}_{q}(\theta)$ and $\mathrm{Im}\,\Psi^{(1)}_{q}(\theta) \neq \mathrm{Im}\,\Psi^{(2)}_{q}(\theta)$.

and

$$-\phi_q = \phi_{-q} \mod 2\pi. \tag{2.15}$$

Having obtained Eqs.(2.14, 2.15), we now attempt to prove the quantization of Berry phase defined in Eq.(1.11). To this end, we consider the Fourier components of $\Psi^{(1)}_q$ and $\Psi^{(2)}_q$, which are related to one another as follows,

$$\psi^{(1)}_{l,-q} = \psi^{(2)}_{l,q}. \tag{2.16}$$

Employing Eq.(2.15) and Eq.(2.16), we compute Berry phase by separating it into two parts, $\gamma = \gamma_1 + \gamma_2$, where

$$\begin{aligned}
\gamma_1 &= i \oint_{\mathrm{BZ}} dq \frac{\sum_l \left(\psi^{(1)*}_{l,q} \frac{\partial \psi^{(1)}_{l,q}}{\partial q} + \psi^{(1)*}_{l,-q} \frac{\partial \psi^{(1)}_{l,-q}}{\partial q} \right)}{\sum_{l'} l' \left(|\psi^{(1)}_{l',q}|^2 + |\psi^{(1)}_{l',-q}|^2 \right)} \\
&= i \oint_{\mathrm{BZ}} dq \frac{\sum_l \psi^{(1)*}_{l,q} \frac{\partial \psi^{(1)}_{l,q}}{\partial q}}{\sum_{l'} l' \left(|\psi^{(1)}_{l',q}|^2 + |\psi^{(1)}_{l',-q}|^2 \right)} \\
&\quad + i \oint_{-\mathrm{BZ}} dq \frac{\sum_l \psi^{(1)*}_{l,q} \frac{\partial \psi^{(1)}_{l,q}}{\partial q}}{\sum_{l'} l' \left(|\psi^{(1)}_{l',q}|^2 + |\psi^{(1)}_{l',-q}|^2 \right)} \\
&= i \left(\oint_{\mathrm{BZ}} - \oint_{\mathrm{BZ}} \right) dq \frac{\sum_l \psi^{(1)*}_{l,q} \frac{\partial \psi^{(1)}_{l,q}}{\partial q}}{\sum_{l'} l' \left(|\psi^{(1)}_{l',q}|^2 + |\psi^{(1)}_{l',-q}|^2 \right)} \\
&= 0,
\end{aligned} \tag{2.17}$$

and

$$\begin{aligned}
\gamma_2 &= \frac{1}{2} \oint_{\mathrm{BZ}} dq \frac{\sum_l l \left(|\psi^{(1)}_{l,q}|^2 + |\psi^{(1)}_{l,-q}|^2 \right)}{\sum_{l'} l' \left(|\psi^{(1)}_{l',q}|^2 + |\psi^{(1)}_{l',-q}|^2 \right)} \frac{\partial \phi_q}{\partial q} \\
&= \frac{1}{2} \oint_{\mathrm{BZ}} dq \frac{\partial \phi_q}{\partial q} = \phi_\pi - \phi_0.
\end{aligned} \tag{2.18}$$

Next, at high-symmetry points $q_{\mathrm{hs}} = 0, \pi$, we find that $\phi_{q_{\mathrm{hs}}} = \phi_{-q_{\mathrm{hs}}}$. Together with Eq.(2.15), we obtain $2\phi_{\mathrm{hs}} = 0 \mod 2\pi$, meaning that

$$\gamma = \phi_\pi - \phi_0 = 0 \text{ or } \pi \mod 2\pi. \tag{2.19}$$

It is at this point that we demonstrate the quantization of nonlinear Berry phase.

In order to state the importance of reflection symmetry for the quantization of nonlinear Berry phase, it is crucial to compare the time-dependence (i.e., the functional form of the wave function) between linear and nonlinear bulk modes. In purely linear systems, the dynamics is captured by the linear Schrödinger equation $H|\Psi(t)\rangle = i\partial_t|\Psi(t)\rangle$, where $|\Psi(t)\rangle = (\Psi^{(1)}, \Psi^{(2)}, \dots, \Psi^{(N)})^\top$ is the time-dependent state vector, $\Psi^{(j)}(t)$ is the j-th wave component, and H is the Hamiltonian represented by a matrix. Using the ansatz $|\Psi_m(t)\rangle = e^{-i\omega_m t}|\Psi_m\rangle$ for the m-th eigenvector, Schrödinger equation reduces to the linear algebraic problem $H|\Psi_m\rangle = \omega_m|\Psi_m\rangle$, where the frequency ω_m serves as the m-th eigenvalue of H. We note that the ansatz adopted for linear Schrödinger equation always implies that the eigenmodes are *sinusoidal in time*. In other words, the eigenmodes are composed of fundamental harmonics only. As a result, all wave components enjoy the nice property by sharing the same time-dependence $\Psi^{(j)}(t) = \Psi^{(j)} e^{-i\omega_m t}$. This conclusion holds true for linear SSH model, where the ratio between the wave components $\Psi^{(1)}(t)/\Psi^{(2)}(t)$ stays as a constant as t changes. However, this nice property no longer holds for nonlinear bulk modes in generalized nonlinear Schrödinger equations. The time-dependence

of nonlinear bulk modes is usually *non-sinusoidal*. Without symmetry constraints, the time-dependence of mode components can be very different, despite that they share the same period. This feature can be easily understood by noticing that the ratio $\Psi^{(j_1)}(t)/\Psi^{(j_2)}(t)$ between any two mode components changes as time t varies. It can be alternatively understood that the ratio between the Fourier components $\psi_l^{(j_1)}/\psi_l^{(j_2)}$ is not constant for different Fourier order l, where ψ_l denotes the l-th Fourier component of Ψ.

With this remarkable difference between linear and nonlinear bulk modes in mind, we are now ready to state the importance of reflection symmetry for the quantization of nonlinear Berry phase γ. Without symmetry constraints, γ in Eq.(1.11) contains all Fourier series that are un-correlated between mode components. Therefore, γ is naturally un-quantized. Reflection symmetry states that these Fourier series are not arbitrary. They are related by $\psi_{l,q}^{(1)}/\psi_{l,-q}^{(2)} \equiv 1$ for all l. The intrinsic relationship between mode components quantize nonlinear Berry phase. We note that in purely linear systems, reflection symmetry quantizes Berry phase in the same way by relating linear wave components via $\psi_{l,q}^{(1)}/\psi_{l,-q}^{(2)} \equiv 1$ for $l = 1$. In summary, reflection symmetry quantizes nonlinear Berry phase by constructing intrinsic relationships between nonlinear wave components.

γ also manifests stability against mode disturbances $\delta\Psi$. We note that the geometric Berry phase γ is defined from *nonlinear bulk modes* Ψ. Thus, the perturbed wave $\Psi + \delta\Psi$ has to be a nonlinear bulk mode in order to have a well-defined γ, otherwise the wave number q is ill-defined, and the adiabatic evolution of q cannot be performed. The motion equations demand that the perturbed nonlinear bulk mode, $\Psi + \delta\Psi$, still respects reflection symmetry. As shown in Eqs.(3) and (6) of the main text, the perturbed nonlinear bulk mode still guarantees the integer-value (i.e., the stability) of nonlinear Berry phase γ, as long as the disturbance $\delta\Psi$ is not large enough to cause topological phase transition.

C. Topological phase transition induced by nonlinearity

Interestingly, there is a topological phase transition in γ induced by increasing the mode amplitudes. The quantized integer value of nonlinear Berry phase jumps from π to 0 as the amplitudes A pass through the phase transition point A_c. This transition amplitude can be determined both analytically and numerically by considering the jump in the integer-valued nonlinear Berry phase γ.

First, we show the topological transition with the increase of amplitudes in an analytic way. We focus on the upper band of the nonlinear SSH model to illustrate the phase transition. By employing reflection symmetry properties, nonlinear Berry phase is reduced as Eq.(2.19): $\gamma = \phi_\pi - \phi_0$, where ϕ_π and ϕ_0 are the relative phases ϕ_q at high-symmetry points $q = 0, \pi$ of the upper-band nonlinear modes. In particular, $\gamma = \pi$ if ϕ_π and ϕ_0 pick different integer values, whereas $\gamma = 0$ if they are equal. In order to determine ϕ_π and ϕ_0 for the upper band, we calculate and compare the nonlinear mode frequencies at high-symmetry points between the upper and lower bands.

We denote the frequency as $\omega(\phi_q, A)$ for the nonlinear mode with the wave number q, relative phase ϕ_q, and amplitude A for later discussions. Therefore, at the high-symmetry point $q = 0$, $\omega(\phi_0 = \pi, A)$ and $\omega(\phi_0 = 0, A)$ denote the mode frequencies with the relative phases $\phi_0 = \pi$ and $\phi_0 = 0$, respectively. At the high-symmetry point $q = \pi$, $\omega(\phi_\pi = \pi, A)$ and $\omega(\phi_\pi = 0, A)$ denote the mode frequencies with the relative phases $\phi_\pi = \pi$ and $\phi_\pi = 0$, respectively. We now compare these frequencies to determine which band they belong to.

Given the nonlinear interactions and the parameters $\epsilon_0 = 1.5$, $c_1 = 0.25$, $c_2 = 0.37$, $d_1 = 0.22$, and $d_2 = 0.02$ in Eq.(7) of the main text, the mode with frequency $\omega(\phi_0 = 0, A)$ always belongs to the upper band for different amplitudes, because $\omega(\phi_0 = 0, A) > \omega(\phi_0 = \pi, A)$. However, the mode with frequency $\omega(\phi_\pi = \pi, A)$ switches bands for different amplitudes. As shown in the inset of Fig.1(e) of the main text, the amplitude dependences of mode frequencies $\omega(\phi_\pi = \pi)$ and $\omega(\phi_\pi = 0)$ are plotted by black and green curves, respectively. For $A < A_c$, the mode with frequency $\omega(\phi_\pi = \pi, A)$ belongs to the upper band because $\omega(\phi_\pi = \pi, A) > \omega(\phi_\pi = 0, A)$. Nonlinear Berry phase picks the non-trivial integer value $\gamma = \phi_\pi - \phi_0 = \pi$. For $A > A_c$, the mode with frequency $\omega(\phi_\pi = 0, A)$ belongs to the upper band since $\omega(\phi_\pi = \pi, A) < \omega(\phi_\pi = 0, A)$. γ picks the trivial value $\gamma = \phi_\pi - \phi_0 = 0$ in this case. γ experiences an interesting topological phase transition induced by the abrupt jump from $\phi_\pi = \pi$ to $\phi_\pi = 0$ as the mode amplitudes A grow beyond the critical value A_c. Meanwhile the mode frequency changes from $\omega(\phi_\pi = \pi, A) > \omega(\phi_\pi = 0, A)$ to $\omega(\phi_\pi = \pi, A) < \omega(\phi_\pi = 0, A)$. A_c is determined by the critical condition when the mode frequencies meet, $\omega(\phi_\pi = \pi, A_c) = \omega(\phi_\pi = 0, A_c)$. Interestingly, nonlinear mode frequencies at high-symmetry points can be derived analytically, as detailed in Sec.III.C. Substituting the analytic results, we numerically solve the topological transition amplitude A_c at which γ experiences a discontinuous jump from π to 0.

We now show numerical results based on Eq.(1.11), where the nonlinear Berry phase $\gamma(A)$ is quantized to be π or 0 and jumps as a step function of the mode amplitudes A. The numerical strategy is given as follows.

Step 1, we identically convert Eq.(1.11) as the summa-

tion of three parts, $\gamma = \gamma^{(1)} + \gamma^{(2)} + \gamma^{(3)}$, where

$$\gamma^{(1)} = \frac{1}{2}\oint_{\mathrm{BZ}} dq \frac{\partial \phi_q}{\partial q},$$

$$\gamma^{(2)} = \frac{1}{2}\oint_{\mathrm{BZ}} dq \frac{\sum_l l\left(|\psi^{(2)}_{l,q}|^2 - |\psi^{(1)}_{l,q}|^2\right)}{\sum_{l'} l'\left(|\psi^{(1)}_{l',q}|^2 + |\psi^{(2)}_{l',q}|^2\right)} \frac{\partial \phi_q}{\partial q},$$

$$\gamma^{(3)} = i\oint_{\mathrm{BZ}} dq \frac{\sum_l \psi^{(1)*}_{l,q}\partial_q \psi^{(1)}_{l,q}}{\sum_{l'} l'\left(|\psi^{(1)}_{l',q}|^2 + |\psi^{(2)}_{l',q}|^2\right)}$$

$$-i\oint_{\mathrm{BZ}} dq \frac{\sum_l \psi^{(2)*}_{l,-q}\partial_q \psi^{(2)}_{l,-q}}{\sum_{l'} l'\left(|\psi^{(1)}_{l',-q}|^2 + |\psi^{(2)}_{l',-q}|^2\right)}. \quad (2.20)$$

Using the reflection-symmetric constraints in Eqs.(2.14, 2.15), it is straightforward to analytically prove that both $\gamma^{(2)}$ and $\gamma^{(3)}$ vanish, and $\gamma^{(1)} = \phi_\pi - \phi_0$ is quantized to the integer value π or 0. As a result, $\gamma(A)$ jumps from $\gamma(A < A_c) = \pi$ to $\gamma(A > A_c) = 0$ at the topological transition amplitude A_c.

Step 2, we numerically compute $\gamma^{(1)}$, $\gamma^{(2)}$, and $\gamma^{(3)}$ by discretizing them as follows,

$$\gamma^{(1)} = \frac{1}{2}\sum_{n=1}^{N_q+1}\left[\phi_{q(n+1)} - \phi_{q(n)}\right],$$

$$\gamma^{(2)} = \frac{1}{2}\sum_{n=1}^{N_q+1} \frac{\sum_l l\left(|\psi^{(2)}_{l,q(n)}|^2 - |\psi^{(1)}_{l,q(n)}|^2\right)}{\sum_{l'} l'\left(|\psi^{(1)}_{l',q(n)}|^2 + |\psi^{(2)}_{l',q(n)}|^2\right)} \times \left[\phi_{q(n+1)} - \phi_{q(n)}\right],$$

$$\gamma^{(3)} = \frac{i}{2}\sum_{n=1}^{N_q+1} \frac{\sum_l \psi^{(1)*}_{l,q(n)}(\psi^{(1)}_{l,q(n+1)} - \psi^{(1)}_{l,q(n)})}{\sum_{l'} l'\left(|\psi^{(1)}_{l',q(n)}|^2 + |\psi^{(2)}_{l',q(n)}|^2\right)}$$

$$-\frac{i}{2}\sum_{n=1}^{N_q+1} \frac{\sum_l \psi^{(2)*}_{l,q(n)}(\psi^{(2)}_{l,q(n-1)} - \psi^{(2)}_{l,q(n)})}{\sum_{l'} l'\left(|\psi^{(1)}_{l',q(n)}|^2 + |\psi^{(2)}_{l',q(n)}|^2\right)}, \quad (2.21)$$

where $q(n)$ denotes the discrete wave number that yields $q(N_q+1) = q(1) + 2\pi$, N_q is the number of discrete wave numbers, $\phi_{q(n)}$ represents the relative phase of mode components, and $\psi^{(j)}_{l,q(n)}$ stands for the l-th Fourier coefficient of the j-th component of the nonlinear mode. Following the instructions of analytic results, our goal is to numerically show that $\gamma^{(1)}$ jumps from one integer value to the other as A passes through A_c, and $\gamma^{(2)} = \gamma^{(3)} = 0$ for all amplitudes.

Step 3, we use the numerical *shooting method* [14–16] to compute nonlinear bulk modes $\Psi_n = (\Psi^{(1)}_q(t), \Psi^{(2)}_q(t))$ with wave number q and mode amplitude A. The algorithmic details of shooting method is elaborated in Sec.III.B. Nonlinear bulk modes are numerically solved for amplitudes ranging from $A_{\min} = 0.003$ to $A_{\max} = 1.8 \approx 2A_c$. We put the mode into the finite lattice with periodic boundary condition, and let it freely oscillate for 10 periods without damping. After that, Fast Fourier Transformation (fft) is performed to calculate the Fourier components $\psi^{(j)}_{l,q(n)}$ for $j = 1, 2$. We truncate the Fourier series to the order $l \leq 10$, and compute $\gamma^{(1,2,3)}$ in Eqs.(2.21).

Step 4, as shown in Fig.2(a), we plot the amplitude dependence of $\gamma^{(1)}$ and $|\gamma^{(2)}| + |\gamma^{(3)}|$ with black and blue lines, respectively. $\gamma^{(1)}$ clearly manifests a discontinuous jump from integer values π to 0 as the amplitude A passes through $A_c = 0.8944$, whereas $|\gamma^{(2)}|$ and $|\gamma^{(3)}|$ are too small to be visible. Fig.2(b) depicts enlarged $|\gamma^{(2)}| + |\gamma^{(3)}|$, which demonstrates their random fluctuations around zero. These fluctuations stem from the tolerance of shooting method that allows the numerical solutions to randomly fluctuate around nonlinear bulk states. In Fig.2(c), γ is quantized and shows the discontinuous jump at the transition amplitude A_c. These numerical results are perfectly in line with what we anticipate from the analytic quantized nonlinear Berry phase.

D. Quantization of nonlinear Berry phase assured by homotopy group $\pi_1(S^1)$

In linear topological band theory, it is known that the quantization of a topological number is assured by homotopy. For example, the winding number is described by the homotopy group $\pi_1(S^1)$. Therefore, it is intriguing to ask what is the homotopy group that ensures the quantization of nonlinear Berry phase.

In purely linear SSH model with the Hamiltonian $H = \vec{h} \cdot \vec{\sigma}$, quantization of Berry phase is assured by the homotopy $\pi_1(S^1)$ that maps from the 1D Brillouin zone to the "magnetic field" $\vec{h}$ constrained on the equator of the Bloch sphere. Likewise, quantization of nonlinear Berry phase is guaranteed by mapping the Brillouin zone $q \in [-\pi, \pi]$ to the "magnetic field $\vec{h}(\Psi)$" constructed from nonlinear bulk modes Ψ. $\vec{h}(\Psi)$ is constrained by reflection symmetry on the $(h_x, h_y, h_z = 0)$ 2D horizontal plane. Thus, the subsequent mapping from 1D Brillouin zone to the closed trajectory $\vec{h}(\Psi)$ in 2D plane picks the quantized integer value.

The aforementioned magnetic field $\vec{h}(\Psi)$ is constructed in the following way. By recalling nonlinear bulk modes $\Psi_q = (\Psi^{(1)}_q(\theta), \Psi^{(2)}_q(\theta + \phi_q))$ in Eq.(2.4), we define the following two functions

$$Z^{(1)}_q = [\Psi^{(1)}_{+q}(-\phi_{+q}) + \Psi^{(1)*}_{-q}(-\phi_{-q})]/2,$$
$$Z^{(2)}_q = [\Psi^{(2)}_{-q}(+\phi_{-q}) + \Psi^{(2)*}_{+q}(+\phi_{+q})]/2. \quad (2.22)$$

In linear SSH model, the wave components are given by $\Psi^{(1)}_q(\theta) = e^{-i\theta}\cos(\theta_q/2)$ and $\Psi^{(2)}_q(\theta) = e^{-i\theta}\sin(\theta_q/2)$. The functions in Eqs.(2.22) are simplified as $Z^{(1)}_q = e^{i\phi_q}\cos(\theta_q/2)$ and $Z^{(2)}_q = e^{i\phi_q}\sin(\theta_q/2)$. Thus, it becomes clear that $\arg(Z^{(1)}_q)$ and $\arg(Z^{(2)}_q)$ can characterize the relative phase ϕ_q between the two linear (nonlinear) wave components, and the ratio $Z^{(1)}_q/Z^{(2)}_q$ captures the relative magnitude of of the two linear (nonlinear) wave

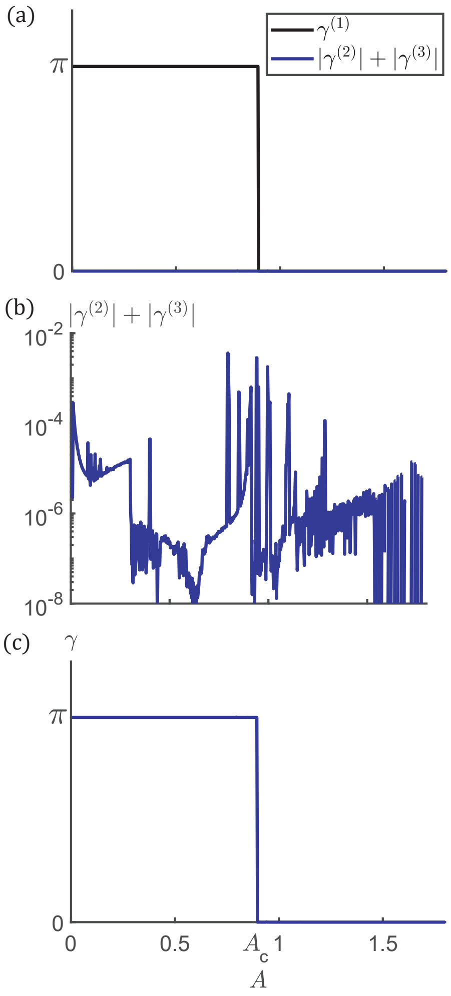

Supplementary Figure 2. Numerical computations of γ, $\gamma^{(1)}$, $\gamma^{(2)}$, and $\gamma^{(3)}$ as the functions of mode amplitudes A ranging from $A = 0.003$ to 1.8. (a) Black and blue lines stand for the amplitude dependences of $\gamma^{(1)}$ and $|\gamma^{(2)}|+|\gamma^{(3)}|$, respectively. A discontinuous jump occurs to $\gamma^{(1)}$ at $A = A_c$, and $|\gamma^{(2)}| + |\gamma^{(3)}|$ is negligible for all amplitudes. (b) Enlarged figure of $|\gamma^{(2)}|+|\gamma^{(3)}|$ states that both $\gamma^{(2)}$ and $\gamma^{(3)}$ randomly fluctuate around 0 due to the numerical error of generating nonlinear bulk states. (c) Quantized nonlinear Berry phase $\gamma = \gamma^{(1)} + \gamma^{(2)} + \gamma^{(3)}$ discontinuously jumps at the critical amplitude $A = A_c$.

components. Next, we construct the three components of the magnetic field $\vec{h}(\Psi)$,

$$h_x(\Psi) = \mathrm{Re}\left[\frac{2Z_q^{(1)}Z_q^{(2)}}{\sqrt{(Z_q^{(1)2}+Z_q^{(2)2})(|Z_q^{(1)}|^2+|Z_q^{(2)}|^2)}}\right],$$

$$h_y(\Psi) = \mathrm{Im}\left[\frac{2Z_q^{(1)}Z_q^{(2)}}{\sqrt{(Z_q^{(1)2}+Z_q^{(2)2})(|Z_q^{(1)}|^2+|Z_q^{(2)}|^2)}}\right],$$

$$h_z(\Psi) = \mathrm{Re}\left[\frac{Z_q^{(1)2}-Z_q^{(2)2}}{Z_q^{(1)2}+Z_q^{(2)2}}\right]. \qquad (2.23)$$

As the wave number traverses the Brillouin zone, h_x and h_y together depict the winding of $\vec{h}(\Psi)$ trajectory around the point $(0, 0, h_z)$, whereas h_z itself describes the magnitude difference between the two nonlinear mode components. In linear SSH model, we can easily obtain $\vec{h} = (\cos\phi_q \sin\theta_q, \sin\phi_q \sin\theta_q, \cos\theta_q)$ from the eigenstates. Without symmetry constraints, $\vec{h}$ is freely movable on a closed trajectory *not* on the equator of the Bloch sphere. Chiral or reflection symmetry demands that $\theta_q = \pi/2$ and the subsequent $\vec{h} = (\cos\phi_q, \sin\phi_q, 0)$ trajectory is constrained on the equator. Likewise, for nonlinear systems without symmetry constraints, $\vec{h}(\Psi)$ can be any trajectory not restricted in the 2D horizontal plane. Reflection symmetry in the nonlinear SSH model demands the wave components to yield Eqs.(2.14,2.15), which in turn offer us the result $Z_q^{(1)} = Z_q^{(2)}$ in Eqs.(2.22). Therefore,

$$\vec{h}(\Psi) = (\cos(\arg Z_q^{(1)}), \sin(\arg Z_q^{(1)}), 0) \qquad (2.24)$$

is in consequence a closed 1D circular trajectory embedded in the 2D horizontal plane. The map from 1D Brillouin zone to $\vec{h}(\Psi)$ is depicted by the homotopy group $\pi_1(S^1) = \mathcal{Z}$ that counts how many times $\vec{h}(\Psi)$ winds around the origin. The topological phase of the nonlinear lattice is described by the winding number $\mathcal{N}$ of $\vec{h}(\Psi)$, which is further captured by

$$\mathcal{N} = (\arg Z_\pi^{(1)} - \arg Z_0^{(1)})/\pi = (\phi_\pi - \phi_0)/\pi, \qquad (2.25)$$

where ϕ_π and ϕ_0 are the relative phases of nonlinear mode components at high-symmetry points. The nonlinear system is topologically non-trivial (trivial) if $\phi_\pi - \phi_0 = \pi$ ($\phi_\pi - \phi_0 = 0$). Interestingly, the system experiences a topological phase transition induced by mode amplitudes as $\phi_\pi - \phi_0$ abruptly changes between 0 and π. This behavior is illustrated in Fig.1(d) of the main text.

With the help of the homotopy group, it is intuitive to understand the quantization fo nonlinear Berry phase under reflection symmetry. The trajectories of $\vec{h}(\Psi)$ constructed from nonlinear bulk modes can compose a 2D surface embedded in 3D space. The homotopy group is to map from the 1D Brillouin zone to $\vec{h}(\Psi)$. Without symmetry constraints, $\vec{h}(\Psi)$ forms a closed path not constrained on the 2D horizontal plane. The continuous mapping from a circle (1D Brillouin zone) to $\vec{h}(\Psi)$ can be continuously deformed into a one-point mapping, and so its homotopy class is trivial. With reflection symmetry constraints, $\vec{h}(\Psi) = (h_x, h_y, 0)$ is restricted on the circular trajectory embedded in 2D horizontal plane. The Brillouin zone circle can be wrapped around the closed trajectory for integer times, which in turn guarantees the quantization of the nonlinear Berry phase in Eq.(1.11).

Concluding remarks: Within the context of topological insulators, homotopies are associated with the linear matrix representations of the Hermitian operators that govern their dynamics, which rightly raises questions as to the application of such principles to nonlinear modes.

For example, a homotopy might be associated in the linear case with how the phase of a vector winds as one traverses a 1D Brillouin Zone. How, then, to obtain a homotopy for a system that lacks a linear vector space? To do so, it is important to recall that in the more general mathematical formalism, there is nothing specific to linearity in defining homotopies. Rather, homotopies are the in-equivalence classes (for example, those indexed by an integer) associated with ways in which one topological space, often a hyper-sphere, can be mapped onto another. These spaces are not in general linear vector spaces. Homotopies may thus be established in spaces of nonlinear modes provided that we have a mapping analogous to the linear case, in which a point in the Brillouin Zone is mapped onto a complex vector (constructed from the wave function).

E. Symmetries and stability of nonlinear bulk states

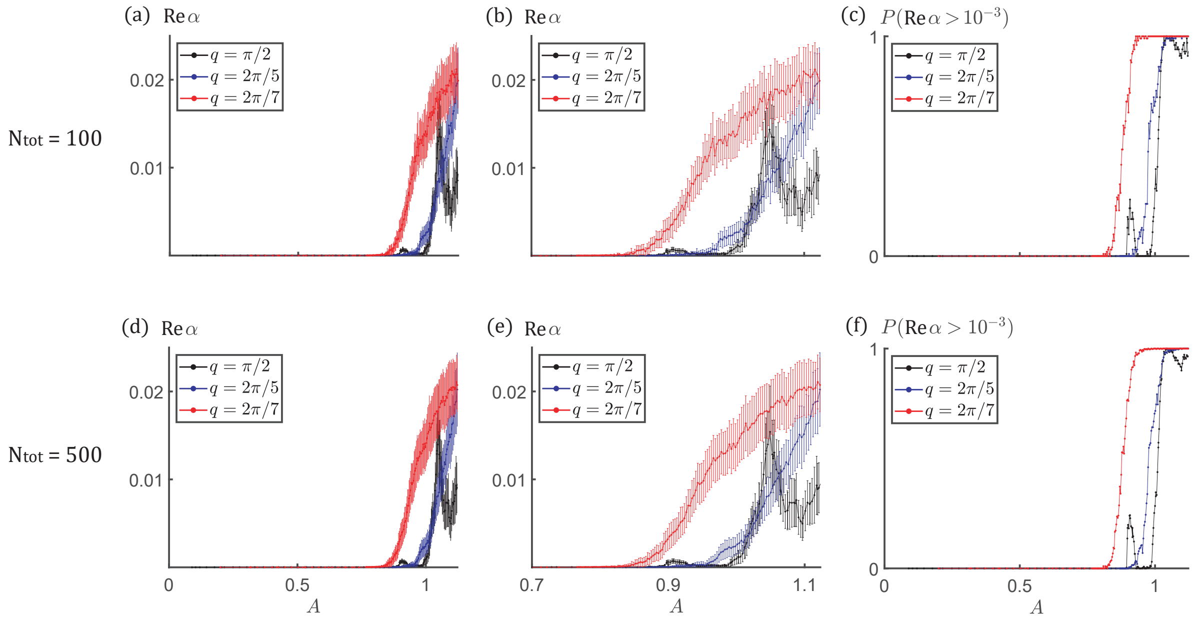

Supplementary Figure 3. Statistics of α for $N_{\text{tot}} = 100$ randomly generated disturbing waves in (a), (b), (c), and $N_{\text{tot}} = 500$ randomly generated disturbing waves in (d), (e), (f), respectively. (a) and (d) Statistics of α. The curve centers are mean values of α, and the error bars are the standard deviations. (b) and (e) Enlarged window for (a) and (d) with the horizontal axis ranged from 0.7 to 1.13. (c) and (f) Percentage of $\mathrm{Re}\,\alpha > 10^{-3}$ for $N_{\text{tot}} = 100$ and 500 α's, respectively.

In this subsection, we discuss the temporal stability of nonlinear bulk modes against wave disturbances. We perform Floquet stability analysis to find new information about the symmetry properties of nonlinear bulk modes. Due to the symmetry constraints, all Floquet exponents are *purely imaginary*, and all nonlinear bulk modes are *marginally stable*. Numerical computation is further performed to confirm marginal stability of nonlinear bulk modes. We find that, as amplitudes rise, numerical results show continuous transition in mode instability that stems from the accumulation of higher-order terms in wave function disturbance. Weak instability can be stabilized by small damping, which is ubiquitous in classical systems.

We initiate the Floquet analysis by recalling the nonlinear motion equations subjected to periodic boundary condition (i.e., Eqs.(1) of the main text), where the on-site potentials are denoted as ϵ_1 and ϵ_2 for A-sites and B-sites, respectively.

$$
\begin{aligned}
i\partial_t \Psi_n^{(1)} &= \epsilon_1 \Psi_n^{(1)} + f_1(\Psi_n^{(1)}, \Psi_n^{(2)}) + f_2(\Psi_n^{(1)}, \Psi_{n-1}^{(2)}),\\
i\partial_t \Psi_n^{(2)} &= \epsilon_2 \Psi_n^{(2)} + f_1(\Psi_n^{(2)}, \Psi_n^{(1)}) + f_2(\Psi_n^{(2)}, \Psi_{n+1}^{(1)}).
\end{aligned}
\tag{2.26}
$$

Reflection symmetry is broken (preserved) when $\epsilon_1 \neq \epsilon_2$ ($\epsilon_1 = \epsilon_2$). We mathematically re-formulate the nonlinear dynamics in Eqs.(2.26) as $\partial_t z(t) = g(z(t))$, where $z(t) = (\ldots, \mathrm{Re}\,\Psi_n^{(1)}, \mathrm{Im}\,\Psi_n^{(1)}, \mathrm{Re}\,\Psi_n^{(2)}, \mathrm{Im}\,\Psi_n^{(2)}, \ldots)$ denotes the field variables, and $g(z)$ can be derived from the motion equations (elaborations in Sec.III.B).

We perturb the nonlinear bulk mode with a small disturbance. The wave function is now given by

$\Psi_n = \Psi_{n,(0)} + \delta\Psi_n$, where $\Psi_{n,(0)} = \Psi_q(\omega t - qn)$ denotes the zeroth-order nonlinear bulk mode, and $\delta\Psi_n = (\delta\Psi_n^{(1)}, \delta\Psi_n^{(2)})^\top$ is the perturbing wave. Hence, z is perturbed from $z_{(0)}$ to $z_{(0)} + \delta z$, where $\delta z = (\ldots, \mathrm{Re}\,\delta\Psi_n^{(1)}, \mathrm{Im}\,\delta\Psi_n^{(1)}, \mathrm{Re}\,\delta\Psi_n^{(2)}, \mathrm{Im}\,\delta\Psi_n^{(2)}, \ldots)^\top$. Since $z_{(0)}$ already satisfies the motion equations, we expand Eqs.(2.26) in orders of δz to have

$$\partial_t \delta z = M(z_{(0)})\delta z + \mathcal{O}(\delta z^2), \tag{2.27}$$

where $M(z) = \partial g(z)/\partial z$ is the monodromy matrix. By dropping $\mathcal{O}(\delta z^2)$ and higher-order terms, Eq.(2.27) is linear in δz, where the monodromy matrix $M(z_{(0)})$ is periodically modulated by $z_{(0)}$ with the period $T = 2\pi/\omega$. It is at this point that the stability analysis is mathematically formulated as a Floquet problem. The stability of nonlinear bulk modes $z_{(0)}$ is captured by the real part of Floquet exponents [16, 24, 25], which we mainly discuss as follows.

Before further devoting into the Floquet exponents, we quickly discuss a property of Eq.(2.27) that stems from reflection symmetry. Reflection symmetry is preserved in the original nonlinear Schrödinger euqations when $\epsilon_1 = \epsilon_2$. Therefore, Eq.(2.27) is invariant under the combined change of $q \to -q$ in $z_{(0)}$, and $\delta\Psi_n \to \delta\Psi_{N-n}$ in δz. Consequently, the Floquet multipliers and eigenvectors have one-to-one correspondence between the two Floquet equations for q and $-q$ in $z_{(0)}$. If $\epsilon_1 \neq \epsilon_2$ and reflection symmetry is broken, then the above-mentioned one-one correspondence is lost.

We now move on to discuss the more interesting property of Floquet exponents. Floquet theorem states that an eigenstate $y_n(t)$ yields $y_n(t+T) = e^{\alpha_n T} y_n(t)$, where α_n is the n-th Floquet exponent. The nonlinear mode is stable if all Floquet exponents yield $|e^{\alpha_n}| < 1$. The mode is unstable if at least one of the exponents yields $|e^{\alpha_n}| > 1$. Finally, the mode is marginally stable if the exponents satisfy $|e^{\alpha_n}| = 1$. Thus, a quantitatively study of the Floquet exponents is needed:

$$\begin{aligned}&\text{Floquet exponents } \{\alpha_n\} \\ &= \text{Eigenvalues of } \int_0^T M(z_{(0)}(t))dt \overset{\text{denote}}{=} \tilde{M},\end{aligned} \tag{2.28}$$

where $\tilde{M}$ is dubbed the time-domain monodromy matrix. So far, we have not yet considered symmetries. Below we show that in our model, $\tilde{M}$ is always real and *anti-symmetric*, and all Floquet exponents must be purely imaginary.

The nonlinear interaction in Eq.(7) of the manuscript has the symmetry property $if_i^*(x,y) = f_i(ix^*, iy^*)$. Given a nonlinear bulk mode $\Psi_n(t) = (\Psi_n^{(1)}(t), \Psi_n^{(2)}(t))^\top$ of wave number q, one can always find a partner solution $\Psi_n(t) = i(\Psi_n^{(1)*}(-t), \Psi_n^{(2)*}(-t))^\top$ of wave number $-q$. Due to the non-degenerate nature of nonlinear bulk modes, the wave form must obey

$$i\Psi_q^{(j)*}(-\theta) = \Psi_{-q}^{(j)}(\theta). \tag{2.29}$$

This property and reflection symmetry together offer us the nice attribute

$$\int_0^{2\pi} \left[\mathrm{Im}\Psi_q^{(j)}(\theta)\right]^n d\theta = \int_0^{2\pi} \left[\mathrm{Re}\Psi_q^{(j')}(\theta)\right]^n d\theta, \tag{2.30}$$

for $n \in \mathcal{Z}^+$, $j \neq j'$, and $j, j' = 1, 2$. As a result, the time-domain monodromy matrix $\tilde{M}$ is always real and anti-symmetric. Floquet exponents are purely imaginary: $\mathrm{Re}\,\alpha_n = 0$, which can be easily understood by re-writing the Floquet problem as $i\partial_t \delta z = (iM)\delta z$, where $(i\tilde{M})^\dagger = i\tilde{M}$ is a Hermitian matrix to host real eigenvalues $i\alpha_n \in \mathcal{R}$. Consequently, all Floquet exponents yield the constraint $|e^{\alpha_n}| = 1$, meaning that in our model, all nonlinear bulk modes are marginally stable against wave function perturbations.

Although Floquet analysis states that all nonlinear bulk modes are marginally stable, higher order terms such as $\mathcal{O}(\delta z^2)$ can slowly accumulate over time and eventually dominate the mode stability. Therefore, we take one step further to investigate the mode stability by a numerical strategy detailed as follows.

Given a nonlinear bulk mode that starts with $z_{(0)}(t = 0)$, we randomly disturb the wave function by δz, where all disturbing wave components yield $|\delta z_i| \leq 10^{-3}A$, and A is the amplitude of the unperturbed nonlinear bulk mode. We allow the mode $z = z_{(0)} + \delta z$ to oscillate/evolve without friction for $t = 100T$, where $T = 2\pi/\omega$ is the period of $z_{(0)}$. During oscillations, the amplification of disturbing wave is equivalently captured by the monodromy matrix,

$$\begin{aligned}\frac{\delta z((n'+1)T)}{\delta z(n'T)} &= \exp\int_{n'T}^{(n'+1)T} M(z_{(0)}(t'))dt' \\ &\overset{\text{denote}}{=} \exp(\tilde{M}(n')).\end{aligned} \tag{2.31}$$

We denote the set of eigenvalues of $\tilde{M}(n')$ as the Floquet exponents $\{\alpha_n\}$, which are computed by performing numerical integration of the monodromy matrix. Mode amplifications are given by the real part of these Floquet exponents, among which the biggest one, $\max\{\mathrm{Re}\,\alpha_n\}$, describes the largest possible amplification. We compute its average value for 100 periods $\mathrm{Re}\,\alpha = \frac{1}{100}\sum^{100}\max\{\mathrm{Re}\,\alpha_n\}$ that depicts the average speed of mode amplification.

We repeat the above strategy by randomly generating N_{tot} mode disturbances. Statistical analysis of $\mathrm{Re}\,\alpha$ is performed by calculating their average and standard deviation. In Fig.3, we plot the statistics of $\mathrm{Re}\,\alpha$ versus the amplitudes of unperturbed nonlinear bulk modes. The statistics manifest several interesting features.

First, the statistics fluctuates around $\mathrm{Re}\,\alpha = \pm 10^{-4}$ for a very long time $t = 100T$ and for amplitudes $A \lesssim 0.9$, which is in good agreement with the marginal stability derived from analytic Floquet analysis. As amplitudes grow beyond $A \gtrsim 1$, modes begin to lose stability due to the accumulation of higher-order effects in wave disturbance. However, due to the numerically computed small Floquet exponents $\mathrm{Re}\,\alpha \sim 0.02$, the perturbed modes are

still not far away from the unperturbed ones, especially for relatively short evolution time $t \sim 10T$. In addition, weak instability can be easily balanced by small damping, which is ubiquitous in classical systems. Second, because Floquet exponents $\mathrm{Re}\,\alpha$ fluctuate around $\mathrm{Re}\,\alpha = \pm 10^{-4}$ for small amplitudes, we set the threshold as 10^{-3} for $\mathrm{Re}\,\alpha$, above which the mode becomes unstable for longer oscillating time. We count the percentage of $\mathrm{Re}\,\alpha > 10^{-3}$ for N_{tot} different initial disturbing waves, and plot the amplitude dependence of $P(\mathrm{Re}\,\alpha > 10^{-3})$. In Figs.3(c) and (f), the $q = 2\pi/5$ and $2\pi/7$ modes manifest continuous phase transition in $P(\mathrm{Re}\,\alpha > 10^{-3})$ for $A \gtrsim 1$. The $q = \pi/2$ mode shows a small jump around $A \sim 0.9$, and then continuously grow to $P(\mathrm{Re}\,\alpha > 10^{-3}) = 100\%$. Finally, we compare the statistics between $N_{\mathrm{tot}} = 100$ in Figs.3(a), (b), (c) and $N_{\mathrm{tot}} = 500$ in Figs.3(d), (e), (f). The standard deviations of $\mathrm{Re}\,\alpha$ are not very different. We think that the reason stems from the accumulated effect of higher-order terms, such as $\mathcal{O}(\delta z^2)$, which are nonlinear in δz. These higher-order terms can enlarge the randomness in disturbing waves. Consequently, the standard deviation and statistics of $\mathrm{Re}\,\alpha$ for $N_{\mathrm{tot}} = 100$ and $N_{\mathrm{tot}} = 500$ are similar.

Some of the nonlinear bulk modes, such as Fig.5(c) and (e), show significant additional components to the pure wave. These components stem from the accumulated effect of higher-order terms in the Floquet analysis of Eq.(2.27) for longer oscillating time.

F. Additional properties when the nonlinear interactions yield $f_i(-x,y) = -f_i(x,-y)$

In the minimal model, the functional forms of nonlinear interactions yield $f_i(-x,y) = -f_i(x,-y)$ (or equivalently, $f_i(-x,-y) = -f_i(x,y)$). Given a nonlinear bulk mode Ψ_q, it is straightforward to prove that $-\Psi_q$ is a nonlinear solution as well. Hence, Ψ_q and $-\Psi_q$ must differ by a phase $\Delta\theta$ only, such that $\Psi_q(\theta+\Delta\theta) = -\Psi_q(\theta)$. We perform the phase shift $\Delta\theta$ twice to have $\Psi_q(\theta+2\Delta\theta) = \Psi_q(\theta)$, which imposes $\Delta\theta = \pi$. Finally, we reach the conclusion

$$\Psi_q(\theta+\pi) = -\Psi_q(\theta). \tag{2.32}$$

G. Symmetry properties of nonlinear bulk modes when $\epsilon_0 = 0$

Having established quantized Berry phase under reflection symmetry, we now search additional properties for vanishing on-site potential, $\epsilon_0 = 0$. When $\epsilon_0 = 0$, the linearized model of Eqs.(1.1) is subjected to an additional symmetry called chiral symmetry [22, 26]. In the linear limit, the interactions are reduced to $f_i(\Psi_n^{(j)}) = c_i \Psi_n^{(j)}$. We convert wave function to wavevector space $\Psi_n^{(j)} = \Psi_\omega^{(j)} e^{i(qn-\omega t)}$ to have the reduced equation of motion, $H\Psi_\omega = \omega\Psi_\omega$, where $H = (c_1 + c_2\cos q)\sigma_x + (c_2 \sin q)\sigma_y$. H is subjected to chiral symmetry, meaning that one can find a symmetry operator $\Pi = \sigma_z$ such that $\Pi^2 = I$, and $\Pi H \Pi^{-1} = -H$. As a result, $H\Pi\Psi_\omega = -\omega\Pi\Psi_\omega$, meaning that the eigenvalues always come in $\pm\omega$ pairs, and the eigenmodes Ψ_ω, $\Psi_{-\omega}$ are related by $\Pi\Psi_\omega = e^{i\phi_q}\Psi_{-\omega}$. This relationship demonstrates the quantization of Berry phase when we evaluate it in the upper band.

We then study the nonlinear model in Eqs.(1.1) with $\epsilon_0 = 0$ and the associated nonlinear modes. In order to have the frequencies of nonlinear modes to appear in $\pm\omega$ pairs, we ask that the nonlinear interactions $f_i(x,y)$ to yield the following constraints:

$$f_i(-x,y) = -f_i(x,-y) = f_i(x,y), \tag{2.33}$$

for $i = 1, 2$. In linear systems, $f_i(x,y)$ is reduced to $f_i(x,y) = c_i y$ and this property is naturally met. However, this property is not naturally satisfied by arbitrary nonlinear functions, and Eqs.(2.33) are the additional constraints for nonlinear interactions. As a result, the system is invariant under the transformation

$$(\Psi_n^{(1)}(\omega t), \Psi_n^{(2)}(\omega t)) \to (-\Psi_n^{(1)}(-\omega t), \Psi_n^{(2)}(-\omega t)). \tag{2.34}$$

Let us consider a nonlinear bulk mode solution Ψ_ω of the upper band with the frequency $\omega > 0$ and wave number q. It yields the following nonlinear differential equation,

$$\begin{aligned} &\Psi_\omega(\theta = \omega t - qn) = (\Psi_q^{(1)}(\theta), \Psi_q^{(2)}(\theta+\phi_q))^\top : \\ &\mathcal{L}(\Psi_\omega; \epsilon_0 = 0) = 0. \end{aligned} \tag{2.35}$$

Referring to Eq.(2.34), it is straightforward to find a "partner solution $\Psi_{-\omega}$" of frequency $-\omega < 0$ and wave number q, that satisfies the nonlinear differential equation,

$$\begin{aligned} &\Psi_{-\omega}(\theta = -\omega t - qn) = (-\Psi_q^{(1)}(\theta), \Psi_q^{(2)}(\theta+\phi_q))^\top : \\ &\mathcal{L}(\Psi_{-\omega}; \epsilon_0 = 0) = \sigma_z \mathcal{L}(\Psi_\omega; \epsilon_0 = 0) = 0. \end{aligned} \tag{2.36}$$

Eq.(2.36) demonstrates that the frequencies of nonlinear bulk modes always appear in $\pm\omega$ pairs. Consequently, $\Psi_{-\omega}$ is the nonlinear bulk mode solution that belongs to the lower band, and the nonlinear band structure is symmetric with respect to $\omega = 0$ axis.

Similar to chiral symmetric models in linear systems [26], the frequencies of nonlinear topological modes are zero, which is illustrated in the minimal model.

III. METHODS OF COMPUTING NONLINEAR BULK MODES

In this section, we introduce the methods of computing nonlinear bulk modes, which are commonly used in solving nonlinear problems. We illustrate these methods by considering the model of Eqs.(1.1) with the nonlinear interactions specified in Eq.(7) of the main text,

$$f_i(x,y) = c_i y + d_i[(\mathrm{Re}\,y)^3 + i(\mathrm{Im}\,y)^3]. \tag{3.1}$$

In the weakly nonlinear regime, the analytic and perturbative *method of multiple-scale* [7, 8, 12, 27] finds nonlinear bulk modes asymptotically, which serves as the

cornerstone of nonlinear modes for higher amplitudes. As the amplitude grows, the system enters into a region where this perturbative technique is unavailable. Instead, the numerical tactic called *shooting method* [14–16] finds nonlinear bulk modes for large amplitudes, and these modes are noticeably different from sinusoidal waves (Figs.1(b) of the main text, and Fig.11(a) and (c) in this supplementary information). In this paper, we combine these two methods, i.e., method of multiple-scale and shooting method, to obtain a series of nonlinear bulk modes for a wide range of amplitudes.

A. Method of multiple-scale: bulk modes in weakly nonlinear regime

First of all, we explore bulk modes in the weakly nonlinear regime. The perturbative approach, namely method of multiple-scale, is useful to solve the frequencies and waveforms of weakly nonlinear bulk modes.

This method is performed by introducing a small book-keeping parameter $\epsilon \ll 1$ that enforces small amplitudes for the bulk modes. Specifically, this parameter is introduced by rewriting d_i as ϵd_i in Eq.(3.1). This method then expands the time derivatives in orders of slow-time derivatives,

$$\frac{d}{dt} = \sum_{l=0}^{\infty} \epsilon^l D_l, \tag{3.2}$$

where $T_{(l)} = \epsilon^l T_{(0)}$ is the l-th order slow time variable, and $D_l = \partial/\partial T_{(l)}$ is the corresponding slow time derivative. Next, the wave function is also expanded in terms of the multiple-scale,

$$\Psi_n = \sum_{l=0}^{\infty} \epsilon^l \Psi_{n,(l)}, \tag{3.3}$$

where $\Psi_{n,(l)} = (\Psi^{(1)}_{n,(l)}, \Psi^{(2)}_{n,(l)})^\top$ is the l-th order wave function. In what follows, we calculate $\Psi_{n,(l=1)}$, which offers us the wave function correction and the frequency correction of the first order. Following Eqs.(3.2, 3.3), we expand the equations of motion by matching all field variables with respect to the order of the book-keeping parameter ϵ. To zeroth-order, the equations of motion are given as

$$L(\Psi_{n,(0)}) = 0, \tag{3.4}$$

where the Linear operator $L(\Psi_n)$ is specified below,

$$L(\Psi_n) = \begin{pmatrix} iD_0\Psi^{(1)}_n - \epsilon_0\Psi^{(1)}_n - c_1\Psi^{(2)}_n - c_2\Psi^{(2)}_{n-1} \\ iD_0\Psi^{(2)}_n - \epsilon_0\Psi^{(2)}_n - c_1\Psi^{(1)}_n - c_2\Psi^{(1)}_{n+1} \end{pmatrix}. \tag{3.5}$$

The solution to the zeroth-order equations is

$$\Psi_{n,(0)} = A(T_{(1)}) e^{iqn - i\omega_{(0)}T_{(0)} - i\theta(T_{(1)})} (1, e^{-i\phi^{(0)}_q})^\top, \tag{3.6}$$

where $\Delta\omega_{(0)} = \omega_{(0)} - \epsilon_0 = \pm\sqrt{c_1^2 + c_2^2 + 2c_1c_2\cos q}$, $\tan\phi^{(0)}_q = -c_2\sin q/(c_1 + c_2\cos q)$, and $\theta = \theta(T_{(1)})$ is the arbitrary phase condition for the bulk modes. We note that this phase is a constant up to the fast time scale $T_{(0)}$ but can depend on the slow time scale $T_{(1)}$. It provides the frequency shift due to the nonlinearities. In what follows, we will focus on computing this frequency shift. To this end, we consider the first-order equations of motion,

$$L(\Psi_{n,(1)}) + \begin{pmatrix} \mathrm{i}D_1\Psi^{(1)}_{n,(0)} - d_1[(\mathrm{Re}\,\Psi^{(2)}_{n,(0)})^3 + \mathrm{i}(\mathrm{Im}\,\Psi^{(2)}_{n,(0)})^3] - d_2[(\mathrm{Re}\,\Psi^{(2)}_{n-1,(0)})^3 + \mathrm{i}(\mathrm{Im}\,\Psi^{(2)}_{n-1,(0)})^3] \\ \mathrm{i}D_1\Psi^{(2)}_{n,(0)} - d_1[(\mathrm{Re}\,\Psi^{(1)}_{n,(0)})^3 + \mathrm{i}(\mathrm{Im}\,\Psi^{(1)}_{n,(0)})^3] - d_2[(\mathrm{Re}\,\Psi^{(1)}_{n+1,(0)})^3 + \mathrm{i}(\mathrm{Im}\,\Psi^{(1)}_{n+1,(0)})^3] \end{pmatrix} = 0. \tag{3.7}$$

The solution of Eq.(3.7), namely the first-order correction of wave function $\Psi_{n,(1)}$, has two components $\Psi_{n,(1)} = \Psi_{n,(1)}(\omega) + \Psi_{n,(1)}(3\omega)$: a fundamental-harmonic part $\Psi_{n,(1)}(\omega)$ and a third-harmonic part $\Psi_{n,(1)}(3\omega)$. We are interested in how the nonlinearities modify the frequencies of the bulk modes, which stem from the secular term generated by the fundamental harmonics. On the other hand, the frequency-tripling part does not contribute to the secular term and the subsequent frequency shift. Hence, we consider the fundamental harmonic part only. The equations of the fundamental part $\Psi_{n,(1)}(\omega)$ are given as follows,

$$L(\Psi_{n,(1)}(\omega)) + e^{i(qn - \omega_{(0)}T_{(0)} - \theta)} \begin{pmatrix} (-D_1A + iAD_1\theta)e^{i\phi^{(0)}_q} - \frac{3}{4}iA^3(d_1 + d_2e^{-iq}) \\ (-D_1A + iAD_1\theta) - \frac{3}{4}iA^3(d_1 + d_2e^{iq})e^{i\phi^{(0)}_q} \end{pmatrix} = 0. \tag{3.8}$$

We want to find $\Psi_{n,(1)}(\omega)$ orthogonal to $\ker(L(\Psi_n))$, which is of the form

$$\Psi_{n,(1)}(\omega) = ia(-1, e^{-i\phi^{(0)}_q})^\top e^{i(qn - \omega_{(0)}T_{(0)} - \theta(T_{(1)}))}, \tag{3.9}$$

where a is a complex number. We use Eq.(3.8) to solve a, D_1A, and $D_1\theta$,

$$\begin{aligned} D_1\theta &= \frac{3A^2}{4}[d_1\cos\phi^{(0)}_q + d_2\cos(\phi^{(0)}_q + q)], \\ D_1A &= 0, \\ a &= \frac{3A^3}{8\Delta\omega_{(0)}}[d_1\sin\phi^{(0)}_q + d_2\sin(\phi^{(0)}_q + q)]. \end{aligned} \tag{3.10}$$

We note that the result $D_1 A = 0$ is natural for undamped systems. In Eqs.(3.10), since $a \in \mathcal{R}$ is real, it is convenient to denote the real quantity $\phi_q^{(1)} = -3A^2[d_1 \sin\phi_q^{(0)} + d_2 \sin(\phi_q^{(0)} + q)]/4\Delta\omega_{(0)}$. To the order $\mathcal{O}(\epsilon^1)$, the bulk mode solution can therefore be simplified as the following compact form,

$$\begin{aligned}\Psi_n &= \Psi_{n,(0)} + \epsilon\Psi_{n,(1)} \\ &= A(1, e^{-i(\phi_q^{(0)}+\epsilon\phi_q^{(1)})})^\top e^{iqn - i(\omega_{(0)}+\epsilon D_1\theta)T_{(0)} + i\epsilon\phi_q^{(1)}/2}.\end{aligned} \tag{3.11}$$

Hence, as the amplitude rises, the relative phase between two wave components changes from $\phi_q^{(0)}$ to $\phi_q^{(0)} + \epsilon\phi_q^{(1)}$.

Next, we compute Berry phase of this first-order corrected wave function Ψ_n in Eqs.(3.11):

$$\begin{aligned}\gamma &= i \oint_{\mathrm{BZ}} dq \, \frac{\langle \Psi_{q,(0)} + \epsilon\Psi_{q,(1)} | \partial_q | \Psi_{q,(0)} + \epsilon\Psi_{q,(1)} \rangle}{\langle \Psi_{q,(0)} + \epsilon\Psi_{q,(1)} | \Psi_{q,(0)} + \epsilon\Psi_{q,(1)} \rangle} \\ &= \frac{1}{2}\int_0^{2\pi} dq \, \partial_q(\phi_q^{(0)} + \epsilon\phi_q^{(1)}),\end{aligned} \tag{3.12}$$

where $\langle \Psi_{q,(0)} + \epsilon\Psi_{q,(1)} | \Psi_{q,(0)} + \epsilon\Psi_{q,(1)} \rangle = 2A^2$ is the normalization factor. At high-symmetry points $q = 0, \pi$, we have $\phi_q^{(0)} = 0$ or π depending on the topological phase of the zeroth-order motion equations. Thus, one always has $\phi_0^{(1)} = \phi_\pi^{(1)} = 0$. Substituting this result in Eq.(3.12), we reduce Berry phase to

$$\begin{aligned}\gamma &= \frac{1}{2}\int_0^{2\pi} dq \, \partial_q(\phi_q^{(0)} + \epsilon\phi_q^{(1)}) \\ &= \frac{1}{2}\int_0^{2\pi} dq \, \partial_q\phi_q^{(0)} = 0 \text{ or } \pi \mod 2\pi,\end{aligned} \tag{3.13}$$

which demonstrates the invariance of quantized Berry phase in the presence of weakly nonlinearity. We note that the above-mentioned analysis is valid when the nonlinear band gap does not close and there is no topological phase transition. If the mode amplitudes are large enough to close the nonlinear band gap and cause topological transitions, a sudden jump will occur to nonlinear Berry phase. In this case, the perturbative analysis is invalid because the denominator in $\phi_q^{(1)}$ is singular: $\lim_{A\to A_c} \Delta\omega_{(0)} \to 0$.

Method of multiple-scale is a trustworthy technique in weakly nonlinear regime by allowing perturbative analysis. It provides nonlinear effects quantitatively, like the frequency shift $D_1\theta$. They help to verify the correctness of other numerical methods in strongly nonlinear regime. The good agreement of the frequency shift in weakly nonlinear regime between method of multiple-scale and shooting method is presented in Fig.4.

B. Shooting method: bulk modes in strongly nonlinear regime

Secondly, we introduce shooting method which numerically computes nonlinear bulk modes of Eqs.(1.1) in strongly nonlinear regime, where the nonlinearities are comparable to the linear interactions and perturbation theory breaks down. We define the $4N \times 1$ vector field $z(t)$ which describes the wave functions of all particles,

$$\begin{aligned}z(t) = \big(&\mathrm{Re}\,\Psi_1^{(1)}, \mathrm{Im}\,\Psi_1^{(1)}, \mathrm{Re}\,\Psi_1^{(2)}, \mathrm{Im}\,\Psi_1^{(2)}, \\ &\ldots, \mathrm{Re}\,\Psi_N^{(1)}, \mathrm{Im}\,\Psi_N^{(1)}, \mathrm{Re}\,\Psi_N^{(2)}, \mathrm{Im}\,\Psi_N^{(2)}\big)^\top.\end{aligned} \tag{3.14}$$

The equation of motion for $z(t)$ is $dz/dt = g(z)$, which in turn gives

$$z(t) = z(0) + \int_0^t g(z(t'))dt', \tag{3.15}$$

where $g(z)$ is a $4N \times 1$ vector derived from the nonlinear equations of motion. Each component is displayed as follows,

$$\begin{aligned}g_{4n-3} &= +\epsilon_0 z_{4n-2} + F_1(z_{4n-0}) + F_2(z_{4n-4}), \\ g_{4n-2} &= -\epsilon_0 z_{4n-3} - F_1(z_{4n-1}) - F_2(z_{4n-5}), \\ g_{4n-1} &= +\epsilon_0 z_{4n-0} + F_1(z_{4n-2}) + F_2(z_{4n+2}), \\ g_{4n-0} &= -\epsilon_0 z_{4n-1} - F_1(z_{4n-3}) - F_2(z_{4n+1}),\end{aligned} \tag{3.16}$$

where $1 \le n \le N$, and $F_i(x) = c_i x + d_i x^3$.

The considered nonlinear wave function at time $t = 0$ reads $z(t = 0)$. It evolves forward in time for T, and then the wave function is given by $z(t = T)$. In general, $z(T) \neq z(0)$ since the considered wave may not be periodic in time. In the rest of this section, we denote the nonlinear mode that starts with $z(0)$ and evolves forward in time for T as $\{z(0), T\}$. We further denote a periodic nonlinear solution as $\{z_\mathrm{p}(0), T_\mathrm{p}\}$, meaning that at $t = 0$ the wave function is $z_\mathrm{p}(t = 0)$ and the mode period is T_p. Thus, it is straightforward to have $z_\mathrm{p}(T_\mathrm{p}) - z_\mathrm{p}(0) = 0$. In order to quantify "how far away" $\{z(0), T\}$ is from $\{z_\mathrm{p}(0), T_\mathrm{p}\}$, we define the "shooting function" $H(z(0), T)$ as follows,

$$H(z(0), T) = z(T) - z(0) = \int_0^T g(z(t))dt. \tag{3.17}$$

$H(z(0), T) \neq 0$ for a temporal aperiodic mode $\{z(0), T\}$, and $H(z_\mathrm{p}(0), T_\mathrm{p}) = 0$ for the periodic solution $\{z_\mathrm{p}(0), T_\mathrm{p}\}$. The smaller the shooting function $H(z(0), T)$ is, the closer $\{z(0), T\}$ is to the periodic solution.

From now on we attempt to find periodic solutions by lowering the shooting function in a recursive algorithm, which is known as shooting method. We start the algorithm with a guessing initial wave function $\{z_1(0), T_1\}$: at $t = 0$, the imported guessing wave is $z_1(t = 0)$ and the imported guessing period is T_1, which means we will evolve $z_1(t = 0)$ forward in time for T_1 to evaluate the shooting function $H(z_1(0), T_1)$. Here, $z_1(t = 0)$ and $T_1 = 2\pi/(\omega_{(0)} + \epsilon D_1\theta)$ are chosen from Eq.(3.11), which is derived by the analytic perturbative method of multiple-scale, and implicitly determines the wave number q. $\{z_1(0), T_1\}$ is not a true periodic solution, and the subsequent shooting function $H(z_1(0), T_1) \neq 0$. In

order to approach the true periodic solution $\{z_{\rm p}(0), T_{\rm p}\}$, we make corrections to $\{z_1(0), T_1\}$ to obtain the second guessing solution $\{z_2(0), T_2\}$. We repeat this process to obtain a series of guessing solutions $\{z_l(0), T_l\}$ $(l \geq 1)$. The corresponding shooting functions $H(z_l(0), T_l)$ slowly converge to zero as l increases.

In the l-th step, the guessing solution is denoted as $\{z_l(0), T_l\}$, and the associated shooting function is $H(z_l(0), T_l) \neq 0$. Therefore, we make corrections $\{\Delta z_l(0), \Delta T_l\}$ to the l-th step guessing solution to obtain the guessing solution of $(l+1)$-th step,

$$\{z_{l+1}(0), T_{l+1}\} = \{z_l(0) + \eta_A^{-1}\Delta z_l(0), T_l + \eta_T^{-1}\Delta T_l\}, \tag{3.18}$$

such that

$$|H(z_{l+1}(0), T_{l+1})| < |H(z_l(0), T_l)|. \tag{3.19}$$

In other words, $H(z_{l+1}(0), T_{l+1})$ is closer to zero than $H(z_l(0), T_l)$. η_A and η_T in Eq.(3.18) are constants greater than 1, which slow down the evolution speed. In order to achieve Eq.(3.19), the correction $\{\Delta z_l(0), \Delta T_l\}$ is determined by the following matrix equation,

$$\begin{aligned} &H(z_{l+1}(0), T_{l+1}) \approx \\ &H(z_l(0), T_l) + \frac{\partial H}{\partial z_l(0)}\Delta z_l(0) + \frac{\partial H}{\partial T_l}\Delta T_l = 0, \end{aligned} \tag{3.20}$$

where the matrices $\partial H/\partial z_l(0)$ and $\partial H/\partial T_l$ will be elaborated on later. By repeating the above process, we generate a sequence of guessing solutions $\{z_l(0), T_l\}$, from which the shooting functions converge to zero,

$$\begin{aligned} &\lim_{l\to\infty} H(z_l(0), T_l) = 0 \\ \Rightarrow &\lim_{l\to\infty}\{z_l(0), T_l\} = \{z_{\rm p}(0), T_{\rm p}\}. \end{aligned} \tag{3.21}$$

As the iteration step l increases, we find the periodic nonlinear solution.

We now compute $\{\Delta z_l(0), \Delta T_l\}$. We first examine the number of variables in $\{\Delta z_l(0), \Delta T_l\}$ versus the number of constraints in Eq.(3.20). At first glance, there are $4N+1$ variables but $4N$ constraints in Eq.(3.20), which means that $\{\Delta z_l(0), \Delta T_l\}$ is indeterminate. However, we note that the solutions we seek are periodic in time. If $\{z_{\rm p}(0), T_{\rm p}\}$ is a periodic solution, so as $\{z_{\rm p}(t \neq 0), T_{\rm p}\}$ for an arbitrary initial time t. In other words, a phase condition has to be imposed to remove this arbitrariness. In our numerics, the phase condition is imposed by letting

$$\Delta z_l(0)|_{4N} = 0, \qquad \forall l \geq 1, \tag{3.22}$$

and then in Eq.(3.20) the numbers of variables and constraints match. Next, we elaborate on the matrices appeared in Eq.(3.20) as follows,

$$\frac{\partial H}{\partial T_l} = g(z_l(T_l)), \tag{3.23}$$

and

$$\frac{\partial H}{\partial z_l(0)} = \zeta(T_l) - I, \tag{3.24}$$

where $\zeta(t) \overset{\rm def}{=} \partial z_l(t)/\partial z_l(0)$, and it is obvious that $\zeta(0) = I$. $\zeta(T_l)$ can be computed in the following way. We find that $d\ln\zeta/dt = M_l$, which in turn gives

$$\zeta(T_l) = \exp\int_0^{T_l} M_l(t)dt, \tag{3.25}$$

where the monodromy matrix M is defined as below

$$M = \partial g(z)/\partial z. \tag{3.26}$$

In our problem, each element of the monodromy matrix M is elucidated as follows,

$$\begin{aligned} M_{4n-3,4n-4} &= +dF_2(x)/dx|_{z_{4n-4}}, \\ M_{4n-3,4n-2} &= +\epsilon_0, \\ M_{4n-3,4n-0} &= +dF_1(x)/dx|_{z_{4n-0}}, \\ M_{4n-2,4n-5} &= -dF_2(x)/dx|_{z_{4n-5}}, \\ M_{4n-2,4n-3} &= -\epsilon_0, \\ M_{4n-2,4n-1} &= -dF_1(x)/dx|_{z_{4n-1}}, \\ M_{4n-1,4n-2} &= +dF_1(x)/dx|_{z_{4n-2}}, \\ M_{4n-1,4n-0} &= +\epsilon_0, \\ M_{4n-1,4n+2} &= +dF_2(x)/dx|_{z_{4n+2}}, \\ M_{4n-0,4n-3} &= -dF_1(x)/dx|_{z_{4n-3}}, \\ M_{4n-0,4n-1} &= -\epsilon_0, \\ M_{4n-0,4n+1} &= -dF_2(x)/dx|_{z_{4n+1}}. \end{aligned} \tag{3.27}$$

In summary, we employ Eqs.(3.22, 3.23, 3.24), to solve $\{\Delta z_l(0), \Delta T_l\}$ in Eq.(3.20) in every iteration step of shooting method.

So far, we have evolved a guessing solution into a periodic solution of certain small amplitude A_1. Our next goal is to find nonlinear periodic solutions of the amplitudes greater than A_1. Let us denote the above well-established nonlinear bulk mode as $\{z_{\rm p}(0; A_1), T_{\rm p}(A_1)\}$. We find nonlinear bulk modes of highers amplitudes by using the following strategy. We rescale the wave function by a uniform factor $1+\xi$ $(\xi \ll 1)$, to initialize shooting method with the new guessing solution,

$$\{z_1(0), T_1\} = \{(1+\xi)z_{\rm p}(0; A_1), T_{\rm p}(A_1)\}. \tag{3.28}$$

Shooting method morphs it into a new periodic solution of the amplitude A_2, which we denote $\{z_{\rm p}(0; A_2), T_{\rm p}(A_2)\}$. We note that A_2 is slightly greater than A_1, but $A_2 \neq (1+\xi)A_1$ because the trial wave function in Eq.(3.28) is not a periodic solution. By repeating this strategy, we get nonlinear bulk modes for a wide range of amplitudes.

Having established the algorithm of shooting method, we now elaborate on the numerical details of all parameters. Two sets of parameters of 1D generalized nonlinear Schrödinger equations are considered in this paper.

In the first set of parameters, the nonlinear model is subjected to reflection symmetry only. The on-site potential ϵ_0 adopted in Eqs.(1.1) are $\epsilon_0 = 1.5$ for main text Fig.1 and Fig.2 and $\epsilon_0 = 8$ for main text Fig.3, and the parameters of nonlinear interactions in Eq.(3.1)

are specified as $c_1 = 0.25$, $c_2 = 0.37$, and $d_1 = 0.22$, $d_2 = 0.02$. We note that the topological attributes are not sensitive to the parameters. These parameters are randomly chosen. In order to numerically solve a nonlinear mode of wave number $q = 2\pi m/N$ $(m, N \in \mathcal{Z})$, we construct a chain of N unit cells composed of classical dimer fields, subjected to periodic boundary condition. Consequently, the wave numbers are rational numbers multiple of 2π. By constructing lattices with different unit cell numbers N, we initialize nonlinear modes with different wave numbers. Since the wave numbers $q = 2\pi \times (2m/2N) = 2\pi(m+N)/N = 2\pi m/N \mod 2\pi$, we further restrict $0 \le m \le N-1$, and $\gcd(m, N) = 1$. We begin shooting method by employing the guessing perturbative solution $\{z_1(0), T_1\}$ in Eq.(3.11), with the period T_1, the wave number $q = 2\pi m/N$, and the small amplitude $A_1 \lesssim 10^{-1}\min(\sqrt{|c_1/d_1|}, \sqrt{|c_2/d_2|}, \sqrt{|(c_1-c_2)/(d_1-d_2)|})$. We simulate the differential equation by executing Runge-Kutta 6th-order [28] (RK6) and converting the time-differential operator $\partial/\partial t$ to the time-step $\Delta t = T_1/N_T$, where $N_T = 1000$. After N_T steps of the motion equations, the wave function should go back to the beginning state if it is a periodic solution. Thus, we compute the shooting function $H(z_1(0), T_1)$ to quantify how far away the wave function is from the periodic solution, and then slowly evolve the wave function towards it. $\eta_A = 300$ and $\eta_T = 10$ are adopted in Eq.(3.18) to slow down the evolution process. In the l-th step of shooting method, the period is evolved to T_l, which in turn asks the time step to be $\Delta t = T_l/N_T$. In other words, we adjust the time difference Δt while keep the number of time steps N_T unchanged throughout the evolution procedure of shooting method. We keep evolving a nonlinear mode before the error of shooting function e reaches the numerical tolerance $e_{\max}$,

$$e \overset{\text{def}}{=} \frac{1}{4N}\sum_{i=1}^{4N} |H_i(z_l(0), T_l)| < e_{\max} = 3\times 10^{-3}, \quad (3.29)$$

where H_i is the ith component of the $4N \times 1$ vector of shooting function, and $e_{\max}$ is the numerical tolerance. In later discussions of this section, we will demonstrate the correspondence between the condition of $e < e_{\max}$ and the stability of nonlinear traveling waves by illustrating a stable mode ($e \ll e_{\max}$), a mode on the verge of stability ($e \lesssim e_{\max}$), and an unstable mode ($e > e_{\max}$) in Fig.5. It is at this point that shooting method returns a periodic nonlinear traveling wave of amplitude A_1 and wave number $q = 2\pi m/N$. The next goal is to find periodic bulk modes of higher amplitudes. To this end, we uniformly rescale the aforementioned wave function by a factor of $1 + \xi$ ($\xi = 3 \times 10^{-3}$), to establish a new shooting procedure. Again, shooting method morphs the trial wave function into a traveling solution of amplitude A_2. We repeat this strategy to obtain a series of nonlinear bulk modes with the given wave number $q = 2\pi m/N$ and a wide range of amplitudes.

Given the wave amplitude A, the nonlinear band structure $\omega = \omega(q \in [0, 2\pi], A)$ is plotted by selecting the frequencies of nonlinear bulk modes when the mode amplitudes A' are within the numerical tolerance,

$$|(A - A')/A| < \xi = 3 \times 10^{-3}. \quad (3.30)$$

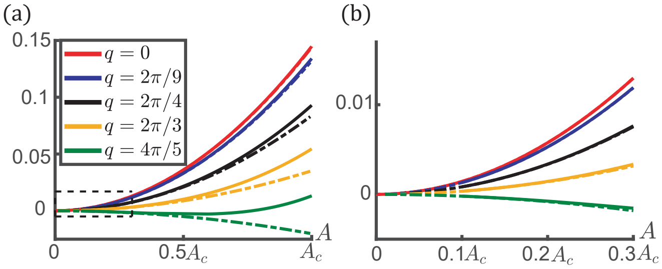

Supplementary Figure 4. Comparing shooting method (solid curves) and method of multiple-scale (dashed curves) on the frequency shift of nonlinear bulk waves. These nonlinear bulk modes start from $A = 0$ in the upper nonlinear band to A_c for a list of wave numbers from $q = 0$ to $4\pi/5$. The model and interaction parameters are depicted by Fig.1 of the main text. Frequency shift computed by shooting method is $\delta\omega(q, A) = \omega(q, A) - \omega(q, A = 0)$, where $\omega(q, A)$ is the frequency of nonlinear bulk mode. Frequency shift obtained by method of multiple-scale is given by $\delta\omega(q, A) = D_1\theta$ in Eqs.(3.10). (a) These two methods agree quite well in weakly nonlinear regime when $A \ll A_c$, while for $A \gtrsim 0.5A_c$, the large deviations demonstrate the breaking down of perturbation theory. (b) Enlarged data for $A \le 0.3A_c$ encircled by the black dashed box in (a).

We have shown nonlinear band structures for a lattice under periodic boundary condition. It is intriguing to ask if it's possible to show a nonlinear band structure for a finite chain under open boundary conditions, where the frequencies of nonlinear edge states are manifest. The idea certainly works for purely linear systems. However, it cannot be extended to strongly nonlinear systems because nonlinear modes that yield open boundary conditions cannot be the linear superposition of nonlinear bulk modes under periodic boundary conditions. To elucidate this problem, we review the band structures of a monatomic 1D linear Schrödinger equation. The bulk state under periodic boundary condition is $e^{iqn - i\omega t}$. Under open boundary conditions, the mode is a standing wave $\Psi_n^{\mathrm{OBC}}(t) = \sin qn \sin\omega t$ to fulfill the boundary conditions. The standing waves and bulk states are convertable since they are linear superposition of each another. Thus, in purely linear systems, the band structures for periodic boundary conditions and open boundary conditions are almost identical except for the addition of localized boundary modes. However, linear superposition is no longer valid for strongly nonlinear systems. Under periodic boundary conditions, the amplitudes of a nonlinear bulk mode are spatially uniform, whereas a nonlinear mode yields vanishing amplitudes at open boundaries. These strongly nonlinear excitations are not

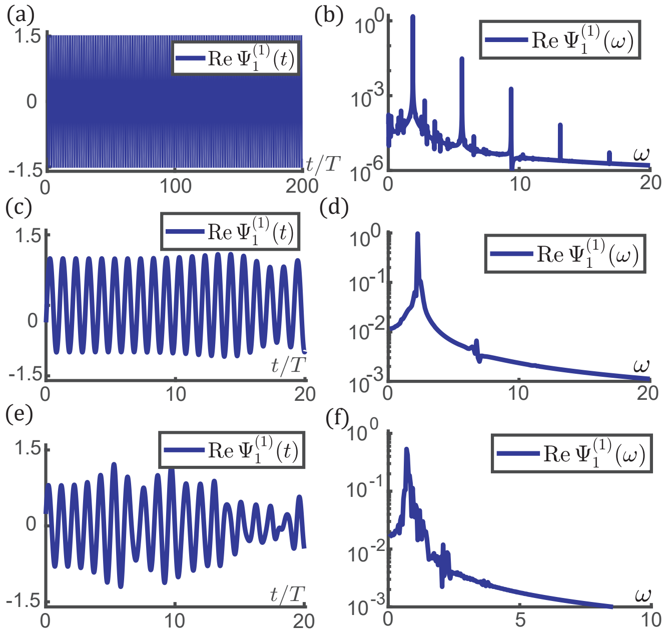

Supplementary Figure 5. Stability analysis of nonlinear bulk modes by performing the algorithm of self-oscillation. (a) A nonlinear bulk mode with the amplitude $A = 1.515$ and wave number $q = 4\pi/5$. The error of shooting function is $e = 10^{-6} \ll e_{\max} = 3 \times 10^{-3}$ (see Eq.(4.1)), which suggests that the mode is stable. To verify our expectation, we initialize the mode from shooting method, and additionally impose a random perturbation $\delta\Psi_n^{(i)}$ on the wave function, where $\mathrm{Re}\,\delta\Psi_n^{(i)}$ and $\mathrm{Im}\,\delta\Psi_n^{(i)}$ are random numbers within 10^{-3}. The mode persists for more than 200 periods without generating other nonlinear modes, which demonstrates mode stability. We note that $A^2 \max(d_1, d_2)/\max(c_1, c_2) = 1.364$, which means the nonlinearities are larger than the linear parts of interactions. (b) The Fourier analysis of (a) after 200 periods provides additional evidence of mode stability. (c) A nonlinear bulk mode with $A = 0.9971$ and $q = 2\pi/9$. The error of shooting function is $e = 2.1 \times 10^{-3} < e_{\max}$, which suggests that the mode is on the verge of stability. We initialize the mode from shooting method without imposing any wave function perturbation. The mode persists for 10 periods with the change of amplitude [8] smaller than 2.3% and is therefore on the verge of stability. (d) The frequency spectrum of the mode in (c) manifests fundamental harmonic and frequency-tripling components. (e) A nonlinear bulk mode with $A = 0.8037$ and $q = 2\pi/21$. The error of shooting function is $e = 7.9 \times 10^{-3} > e_{\max}$, which indicates that the mode is unstable. The mode initialized by shooting method persists in 5 periods of oscillation, and it quickly exhibits mode instability by producing other nonlinear modes. (f) The frequency profile of (e) demonstrates the emergence of other Fourier components, which identifies mode instability.

linear superposition of each another. Furthermore, it remains unknown whether nonlinear standing modes under open boundary conditions can be viewed as "bulk modes" because wave number may not be well-defined. Consequently, the nonlinear mode frequencies for open boundaries may largely differ from that under periodic boundary conditions.

We now turn to discuss the stability analysis of nonlinear bulk modes. The stability analysis of nonlinear modes [8, 16] is to measure how many periods they persist in an undriven, undamped lattice before falling apart. According to Ref. [8], the mode is considered stable if an instability does not occur within 10 periods of oscillation. In order to perform the stability analysis for a nonlinear bulk mode with the wave number $q = 2\pi m/N$, we construct a lattice that comprises N dimer unit cells and is subjected to periodic boundary condition. We establish a nonlinear bulk mode obtained from shooting method. After letting the mode to oscillate by itself for more than 10 periods, Fourier analysis is applied to characterize whether the mode experiences instability and falls apart to other nonlinear modes. In Fig.5, we exemplify three different bulk modes to verify the correspondence between Eq.(3.29) and the mode stability. Hence, all nonlinear bulk modes depicted in the nonlinear band structures of main text Fig.1(e) are considered stable, and they fulfill the criteria of the nonlinear extension of adiabatic theorem [17–20].

In the second set of parameters, $c_1 = 0.25$, $c_2 = 0.37$, $d_1 = 0.22$, $d_2 = 0.02$ are carried over, while ϵ_0 is now set to zero. Nonlinear bulk modes always appear in $\pm\omega$ pairs. Similar to the linear counterpart in which chiral symmetry [26] is present, the frequencies of nonlinear topological edge modes are zero in the second case.

C. Topological transition amplitude A_c: calculating nonlinear bulk modes at high-symmetry points

In this subsection, we solve nonlinear bulk modes at high-symmetry points when $q_{\mathrm{hs}} = 0, \pi$. This allows us to numerically find the topological transition amplitude A_c as well as the band-touching frequency ω.

We denote the nonlinear bulk modes at high-symmetry points as Ψ_{hs}. According to Eq.(2.15), the relative phase at high-symmetry points are $\phi_{\mathrm{hs}} = 0$ or π. The motion equation of Ψ_{hs} is greatly simplified by employing Eqs.(2.14, 2.32),

$$(i\omega\partial_\theta - \epsilon_0)\Psi_{\mathrm{hs}}^{(j)} = e^{i\phi_{\mathrm{hs}}} f_1(\Psi_{\mathrm{hs}}^{(j')}, \Psi_{\mathrm{hs}}^{(j)}) + e^{i(q_{\mathrm{hs}}+\phi_{\mathrm{hs}})} f_2(\Psi_{\mathrm{hs}}^{(j')}, \Psi_{\mathrm{hs}}^{(j)}), \quad (3.31)$$

for $j = 1, 2$. The nonlinear interactions are adopted from Eq.(3.1). By solving Eq.(3.31), $(\mathrm{Re}\,\Psi_{\mathrm{hs}}^{(j)}, \mathrm{Im}\,\Psi_{\mathrm{hs}}^{(j)})$ yield the trajectory,

$$[(\mathrm{Re}\,\Psi_{\mathrm{hs}}^{(j)})^2 - x_0]^2 + [(\mathrm{Im}\,\Psi_{\mathrm{hs}}^{(j)})^2 - x_0]^2 = R^2, \quad (3.32)$$

where R^2 is the constant of integration which quantifies the "radius" of the trajectory, and

$$x_0 = -\frac{\epsilon_0 + e^{i\phi_{\mathrm{hs}}} c_1 + e^{i(q_{\mathrm{hs}}+\phi_{\mathrm{hs}})} c_2}{e^{i\phi_{\mathrm{hs}}} d_1 + e^{i(q_{\mathrm{hs}}+\phi_{\mathrm{hs}})} d_2}. \quad (3.33)$$

In the linear limit, the trajectory simply reduces to a circle, which is in perfect agreement with linear models.

Based on Eq.(3.32), we further obtain the mode frequencies:

$$\omega(q_{\rm hs},\phi_{\rm hs}) = \frac{\pi}{2}\left[\int_0^A \frac{du/|d_1+e^{iq_{\rm hs}}d_2|}{y(u)\sqrt{|y(u)+e^{i\phi_{\rm hs}}x_0|}}\right]^{-1}, \quad (3.34)$$

where A is the mode amplitude, and

$$y(u) = \sqrt{x_0^2+(A^2-x_0)^2-(u^2-x_0)^2}. \quad (3.35)$$

A quick check of the above result is to perform the intergration in the weakly nonlinear regime when $A \ll \sqrt{|x_0|}$. Eq.(3.34) reduces to $\omega = |\epsilon_0 + e^{i\phi_{\rm hs}}c_1 + e^{i(q_{\rm hs}+\phi_{\rm hs})}c_2|$, which is in line with the high-symmetry eigenfrequencies in the linear models. In this paper, the numerical parameters we adopt yield $\epsilon_0, c_1, c_2, d_1, d_2 > 0$, and $c_1 < c_2$, $d_1 > d_2$. Thus, the topological phase transition occurs when the frequencies of nonlinear modes merge at the critical amplitude A_c when

$$\omega(\phi_\pi = 0, A_c) = \omega(\phi_\pi = \pi, A_c). \quad (3.36)$$

This transition amplitude A_c can be obtained by numerically solving the above equation, which is shown in Fig.6(b). In linear SSH model, the topological transition point occurs at the frequency $\omega(\phi_\pi = 0) = \omega(\phi_\pi = \pi) = \epsilon_0$, which is in perfect agreement with the frequency of topological boundary modes, $\omega_{\rm T} = \epsilon_0$. Thus, the frequency of topological modes is always separated from the bulk bands unless topological transition is reached. Unlike linear models, the topological transition of the nonlinear system occurs at the frequency $\omega(\phi_\pi = 0, A_c) = \omega(\phi_\pi = \pi, A_c) = (1 + 3\times 10^{-4})\epsilon_0$, which is slightly different from ϵ_0 (Fig.1(d) of the main text). The small rectification of band-touching frequency stems from the coupling between higher-order and fundamental bulk mode components. On the other hand, the frequencies of nonlinear topological modes $\omega_{\rm T} = \epsilon_0$ are approximately solved by truncating the motion equations to the fundamental harmonics. Thus, if we consider all couplings among higher-order harmonics, the frequencies of topological modes are rectified as well to stay in the bandgap and are thus separated from nonlinear bulk bands. In addition to the distinguishable frequencies, the amplitudes of each site are remarkably different between nonlinear bulk and edge modes. In nonlinear bulk modes, the amplitudes are equal for A and B-sites, whereas the amplitudes of B-sites are negligible compared to A-sites for topological edge modes. When nonlinear topological modes reach the critical amplitude and penetrate infinitely into the lattice, this *nonlinear* mode cannot be decomposed as the superposition of two nonlinear bulk modes. This is in sharp contrast to linear systems in which at the transition point, topological modes can be represented as the superposition of two bulk modes. Thus, nonlinear topological boundary modes are separated from bulk modes, in the sense that they cannot be continuously deformed into one another.

IV. NONLINEAR TOPOLOGICAL EDGE MODES

In this section, we study nonlinear topological edge modes based on the model of Eqs.(1.1) with the interactions specified in Eq.(3.1). To have topological edge modes, we consider a semi-infinite lattice subjected to the open boundary condition

$$\begin{aligned}
&i\partial_t\Psi_n^{(1)} = \epsilon_0\Psi_n^{(1)} + f_1(\Psi_n^{(1)},\Psi_n^{(2)}) + f_2(\Psi_n^{(1)},\Psi_{n-1}^{(2)}),\\
&i\partial_t\Psi_n^{(2)} = \epsilon_0\Psi_n^{(2)} + f_1(\Psi_n^{(2)},\Psi_n^{(1)}) + f_2(\Psi_n^{(2)},\Psi_{n+1}^{(1)}),\\
&\text{for} \quad n\geq 1, \quad \text{and} \quad \Psi_0^{(2)} = 0.
\end{aligned}\quad (4.1)$$

In subsections 1 and 2, we investigate topological edge modes for the model with $\epsilon_0 \neq 0$. In subsection 3, we explore topological modes for the vanishing on-site potential $\epsilon_0 = 0$. The parameters we consider yield $0 < c_1 < c_2$, $d_1 > d_2 > 0$.

A. Method of multiple-scale: topological edge modes for the $\epsilon_0 \neq 0$ case in weakly nonlinear regime

Based on the numerical simulation and qualitative analysis presented in the main text, it is demonstrated that the frequency of topological edge mode is $\omega_{\rm T} = \epsilon_0$ and is independent of the mode amplitude A. This result is in sharp contrast to the amplitude-dependent frequencies of nonlinear bulk modes. Here in weakly nonlinear regime, we quantitatively exhibit this result by employing the method of multiple-scale. Meanwhile, we analytically study the stability of nonlinear topological boundary modes in weakly nonlinear regime. To this end, We consider the equations of motion with the addition of a damping term,

$$\begin{aligned}
&i(\partial_t+\eta)\Psi_n^{(1)} = \epsilon_0\Psi_n^{(1)} + f_1(\Psi_n^{(1)},\Psi_n^{(2)}) + f_2(\Psi_n^{(1)},\Psi_{n-1}^{(2)})\\
&i(\partial_t+\eta)\Psi_n^{(2)} = \epsilon_0\Psi_n^{(2)} + f_1(\Psi_n^{(2)},\Psi_n^{(1)}) + f_2(\Psi_n^{(2)},\Psi_{n+1}^{(1)}),
\end{aligned}\quad (4.2)$$

where η is the damping coefficient that can be realized in classical structures, such as the *RLC* circuit in Fig.5 of the main text.

Method of multiple-scale introduces a book-keeping small parameter $\epsilon \ll 1$ that enforces small amplitudes for the edge modes, which is practically realized by rewriting η and d_i as $\epsilon\eta$ and ϵd_i, respectively. The time derivative and the wave function are expanded in orders of ϵ (see Eqs.(3.2, 3.3)). We expand the equations of motion and match them in orders of ϵ. Following these notations, it is straightforward to expand the temporal evolution of the mode amplitude as

$$\partial_t A = D_0A + \epsilon D_1 A + \epsilon^2 D_2 A + \ldots. \quad (4.3)$$

In particular, $\partial_t A < 0$ if the mode is stable, whereas for $\partial_t A > 0$ the nonlinear mode is unstable. Below, we expand the motion equations to order ϵ^2 to figure out the

mode stability in Eq.(4.3) by computing D_0A, D_1A, and D_2A.

The zeroth-order equations of motion are presented by Eq.(3.4) respecting the open boundary condition $\Psi^{(2)}_{0,(0)} = 0$. The zeroth-order solution reads

$$\Psi_{n,(0)} = (-\kappa)^{n-1} A(T_{(1)}, T_{(2)}) \times e^{-i\omega_{(0)}T_{(0)} - i\theta(T_{(1)},T_{(2)})}(1,0)^\top, \tag{4.4}$$

where $\kappa = c_1/c_2$ denotes the spatial decay rate, and $\omega_{(0)} = \epsilon_0$ is the zeroth-order frequency of the topological mode. $A(T_{(1)}, T_{(2)})$ and $\theta(T_{(1)}, T_{(2)})$ are independent of $T_{(0)}$ but can depend on $T_{(1)}$ and $T_{(2)}$. In particular, $\epsilon D_1\theta$ and $\epsilon^2 D_2\theta$ provide the first and second-order corrections of the mode frequency. $\epsilon D_1 A$ and $\epsilon^2 D_2 A$ manifest the first and second-order nonlinear effects in the mode stability, as shown in Eq.(4.3) above. They are determined via the expansions of motion equations. The first-order expansion of motion equations is given by

$$\begin{aligned} & L(\Psi_{n,(1)}) + i(D_1 + \eta)\Psi_{n,(0)} - \\ & d_1 \begin{pmatrix} (\mathrm{Re}\,\Psi^{(2)}_{n,(0)})^3 + \mathrm{i}(\mathrm{Im}\,\Psi^{(2)}_{n,(0)})^3 \\ (\mathrm{Re}\,\Psi^{(1)}_{n,(0)})^3 + \mathrm{i}(\mathrm{Im}\,\Psi^{(1)}_{n,(0)})^3 \end{pmatrix} - \\ & d_2 \begin{pmatrix} (\mathrm{Re}\,\Psi^{(2)}_{n-1,(0)})^3 + \mathrm{i}(\mathrm{Im}\,\Psi^{(2)}_{n-1,(0)})^3 \\ (\mathrm{Re}\,\Psi^{(1)}_{n+1,(0)})^3 + \mathrm{i}(\mathrm{Im}\,\Psi^{(1)}_{n+1,(0)})^3 \end{pmatrix} = 0 \end{aligned} \tag{4.5}$$

subjected to the open boundary condition $\Psi^{(2)}_{0,(1)} = 0$. There are two parts in this first-order correction of the wave function, namely the fundamental harmonic part $\Psi_{n,(1)}(\omega_\mathrm{T})$ and the frequency-tripling part $\Psi_{n,(1)}(3\omega_\mathrm{T})$: $\Psi_{n,(1)} = \Psi_{n,(1)}(\omega_\mathrm{T}) + \Psi_{n,(1)}(3\omega_\mathrm{T})$. We are interested in the frequency correction and amplitude temporal evolution due to the nonlinearities, which stems from the secular term generated by the fundamental harmonic part. The fundamental harmonics is given by $\Psi_{n,(1)} = (\Psi^{(1)}_{n,(1)}, 0)^\top$, where

$$\Psi^{(1)}_{n,(1)} = (-\kappa)^{n-1} \frac{1-\kappa^{2n-2}}{1-\kappa^2} \frac{3(d_1 - d_2\kappa^3)A^3}{4c_1} e^{-i(\omega_{(0)}T_{(0)} + \theta)} \tag{4.6}$$

and

$$D_1\theta = 0, \qquad D_1 A = -\eta A. \tag{4.7}$$

The second order expansion of the motion equations are given by

$$\begin{aligned} & L(\Psi_{n,(2)}) + iD_2\Psi_{n,(0)} + i(D_1 + \eta)\Psi_{n,(1)} - \\ & 3d_1 \begin{pmatrix} (\mathrm{Re}\,\Psi^{(2)}_{n,(0)})^2 \mathrm{Re}\,\Psi^{(2)}_{n,(1)} \\ (\mathrm{Re}\,\Psi^{(1)}_{n,(0)})^2 \mathrm{Re}\,\Psi^{(1)}_{n,(1)} \end{pmatrix} - \\ & 3id_1 \begin{pmatrix} (\mathrm{Im}\,\Psi^{(2)}_{n,(0)})^2 \mathrm{Im}\,\Psi^{(2)}_{n,(1)} \\ (\mathrm{Im}\,\Psi^{(1)}_{n,(0)})^2 \mathrm{Im}\,\Psi^{(1)}_{n,(1)} \end{pmatrix} - \\ & 3d_2 \begin{pmatrix} (\mathrm{Re}\,\Psi^{(2)}_{n-1,(0)})^2 \mathrm{Re}\,\Psi^{(2)}_{n-1,(1)} \\ (\mathrm{Re}\,\Psi^{(1)}_{n+1,(0)})^2 \mathrm{Re}\,\Psi^{(1)}_{n+1,(1)} \end{pmatrix} - \\ & 3id_2 \begin{pmatrix} (\mathrm{Im}\,\Psi^{(2)}_{n-1,(0)})^2 \mathrm{Im}\,\Psi^{(2)}_{n-1,(1)} \\ (\mathrm{Im}\,\Psi^{(1)}_{n+1,(0)})^2 \mathrm{Im}\,\Psi^{(1)}_{n+1,(1)} \end{pmatrix} = 0 \end{aligned} \tag{4.8}$$

subjected to the open boundary condition $\Psi^{(2)}_{0,(2)} = 0$. The vanishing secular terms demand the second-order solution to yield $\Psi_{n,(2)} = (\Psi^{(1)}_{n,(2)}, 0)^\top$, where

$$\begin{aligned} \Psi^{(1)}_{n,(2)} = (-\kappa)^{n-1} \Big[& \frac{1-\kappa^{2n-2}}{1-\kappa^2}(d_1 - d_2\kappa^3) - \\ & \frac{1-\kappa^{4n-4}}{1-\kappa^4}(d_1 - d_2\kappa^5) \Big] \frac{27(d_1 - d_2\kappa^3)A^5}{16c_1c_2\kappa(1-\kappa^2)} e^{-i\omega_{(0)}T_{(0)} - i\theta} \end{aligned} \tag{4.9}$$

and

$$D_2\theta = 0, \quad D_2 A = \eta \frac{1-\kappa^{2n-2}}{1-\kappa^2} \frac{3(d_1 - d_2\kappa^3)}{2c_1} A^3. \tag{4.10}$$

The results in Eqs.(4.6, 4.9) indicate that the frequency of the topological mode remains unchanged in the presence of nonlinearity and is independent of the mode amplitude. This is in line with the qualitative analysis and numerical computations of nonlinear topological modes in the main text. Also, the zeroth, first, and second-order wave functions exponentially decay in space, which means that the localization of the topological mode is also insensitive to nonlinearities. In other words, topological boundary modes manifest (spatial) stability against nonlinearities. These results echo with Eq.(3.13), the quantized integer Berry phase in the weakly nonlinear regime. Finally, if Berry phase in purely linear regime takes the trivial integer value $\gamma = 0$, then topological evanescent states remain absent in the weakly nonlinear regime.

We now discuss the temporal stability of nonlinear topological boundary modes. According to Eq.(4.3), the topological modes show stability (instability) for $\partial_t A < 0$ ($\partial_t A > 0$), which means that the modes are stable as long as the amplitudes yield $A < A_{c,\mathrm{stability}}$. Collecting the results in Eqs.(4.7, 4.10), the critical amplitude $A_{c,\mathrm{stability}}$ is given by

$$A_{c,\mathrm{stability}} = \sqrt{\frac{2c_1(1-\kappa^2)}{3\epsilon(d_1 - d_2\kappa^3)}}. \tag{4.11}$$

We comment on this result. First, it is derived using the perturbative method of multiple scale, which

is valid in the weakly nonlinear regime. This result qualitatively describes the stability of nonlinear topological modes in the strongly nonlinear regime. Second, the critical stability amplitude $A_{c,\text{stability}}$ and the topological transition amplitude $A_{c,\text{topological}} = \sqrt{-4(c_2 - c_1)/3\epsilon(d_2 - d_1)}$ are equal in the weakly nonlinear regime: $A_{c,\text{stability}}/A_{c,\text{topological}} = 1 + \mathcal{O}(d_i A_{c,\text{topological}}^2/c_i)$. Therefore, all weakly nonlinear topological boundary modes are stable as long as the system stays in the topological phase.

It is worth emphasizing that the weakly nonlinear topological modes derived in Eqs.(4.6) also fulfill Eq.(4.14), which is the recursion relation of the spatial profile of topological edge mode amplitudes in strongly nonlinear regime. These results from method of multiple-scale are in perfect agreement with the methods in strongly nonlinear regime discussed below.

B. Harmonic balance method: topological edge modes for the $\epsilon_0 \neq 0$ case in strongly nonlinear regime

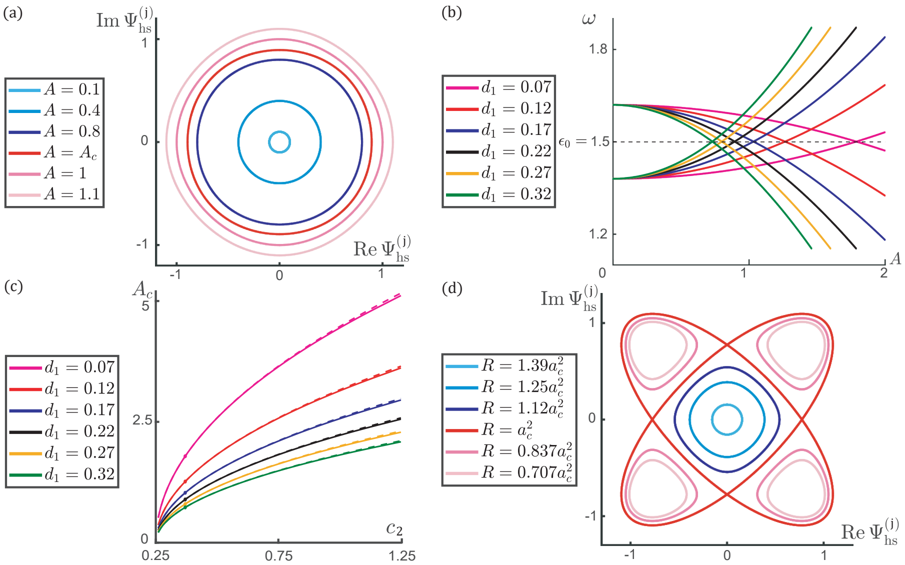

Supplementary Figure 6. The orbits of the field variables at high symmetry points, and the corresponding topological transition amplitudes. (a) In the model with the parameters enumerated in Fig.1 of the main text, we plot the $(\mathrm{Re}\,\Psi_{\text{hs}}^{(j)}, \mathrm{Im}\,\Psi_{\text{hs}}^{(j)})$ trajectories for nonlinear bulk modes at high-symmetry points with a set of amplitudes ranging from $A = 0.1$ to 1.1. The trajectories are noticeably different from regular circles for $A \gtrsim A_c$. (b) Transition amplitude A_c is numerically solved by Eq.(3.36). Here, we exemplify these numerical solutions by varying d_1 from 0.07 to 0.32, where the transition amplitude $A_c = 0.8944$ for $d_1 = 0.22$ is depicted by the intersection of black curves. All illustrated transition amplitudes occur at the merging frequency $\omega(\phi_\pi = 0, A_c) = \omega(\phi_\pi = \pi, A_c) = \epsilon_0$. (c) The nice agreement between numerically solved A_c (solid curves) and its estimation $A_c \approx \sqrt{4/3}a_c$ (dashed curves), where c_2 varies from 0.26 to 1.25, and d_1 varies from 0.07 to 0.32. The transition amplitudes in (b) are marked by colored dots here. We note that the estimations of A_c are worse for $c_2 \gtrsim 1.25$, which is much greater than $c_2 = 0.37$ in our model. (d) In the second case, all interaction parameters are the same as (a) except that $\epsilon_0 = 0$. We plot multiple $(\mathrm{Re}\,\Psi_{\text{hs}}^{(j)}, \mathrm{Im}\,\Psi_{\text{hs}}^{(j)})$ trajectories with the "constant of integration" R that varies from $1.39a_c^2$ to $0.707a_c^2$ (R is defined in Eq.(3.32)). Blue and red curves describe nonlinear modes before and after instability occurs, respectively. The instability happens at $R = a_c^2$ which corresponds to mode amplitude $\max|\mathrm{Re}\,\Psi_1^{(1)}| = a_c$. Above the instability point (i.e., $R < a_c^2$ and $\max|\mathrm{Re}\,\Psi_1^{(1)}| > a_c$), wave functions oscillate around new equilibrium positions.

We now employ the harmonic balance method [29] to study topological edge modes in strongly nonlinear regime. Since the mode is periodic in time, it can be expressed as the Fourier series $\Psi_n = \sum_l \psi_{l,n} e^{-il\omega_{\text{T}}t}$. We take the approximation by truncating the wave function to the fundamental harmonics,

$$\Psi_n \approx \psi_{1,n} e^{-i\omega_{\text{T}}t} + \psi_{-1,n} e^{i\omega_{\text{T}}t} = \frac{1}{2}\begin{pmatrix} \alpha_n^{(1)} + i\alpha_n^{(2)} \\ \beta_n^{(1)} + i\beta_n^{(2)} \end{pmatrix} e^{-i\omega_{\text{T}}t} + \frac{1}{2}\begin{pmatrix} \alpha_n^{(1)*} + i\alpha_n^{(2)*} \\ \beta_n^{(1)*} + i\beta_n^{(2)*} \end{pmatrix} e^{i\omega_{\text{T}}t}, \tag{4.12}$$

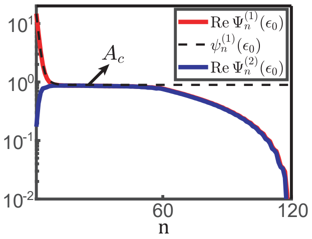

Supplementary Figure 7. Here we plot the entire spatial profile of the $\omega = \epsilon_0$ Fourier component of the nonlinear topological excitation in Fig.3 of the main text to complement the results. The plateau reaches site $n \sim 60$ before falling apart to other nonlinear modes. Fourier analysis is performed by considering the excitations from $200T$ to $400T$.

where $\alpha_n = (\alpha_n^{(1)}, \alpha_n^{(2)})^\top$ and $\beta_n = (\beta_n^{(1)}, \beta_n^{(2)})^\top$ are 2×1 complex vectors parametrizing $\psi_{\pm 1,n}$. Hence, the real and imaginary parts of the wave functions can be expressed as

$$\begin{aligned}
\operatorname{Re}\Psi_n^{(1)} &= \frac{1}{2}\left(\alpha_n^{(1)} e^{-i\omega_{\mathrm{T}} t} + \alpha_n^{(1)*} e^{i\omega_{\mathrm{T}} t}\right), \\
\operatorname{Im}\Psi_n^{(1)} &= \frac{1}{2}\left(\alpha_n^{(2)} e^{-i\omega_{\mathrm{T}} t} + \alpha_n^{(2)*} e^{i\omega_{\mathrm{T}} t}\right), \\
\operatorname{Re}\Psi_n^{(2)} &= \frac{1}{2}\left(\beta_n^{(1)} e^{-i\omega_{\mathrm{T}} t} + \beta_n^{(1)*} e^{i\omega_{\mathrm{T}} t}\right), \\
\operatorname{Im}\Psi_n^{(2)} &= \frac{1}{2}\left(\beta_n^{(2)} e^{-i\omega_{\mathrm{T}} t} + \beta_n^{(2)*} e^{i\omega_{\mathrm{T}} t}\right). \qquad (4.13)
\end{aligned}$$

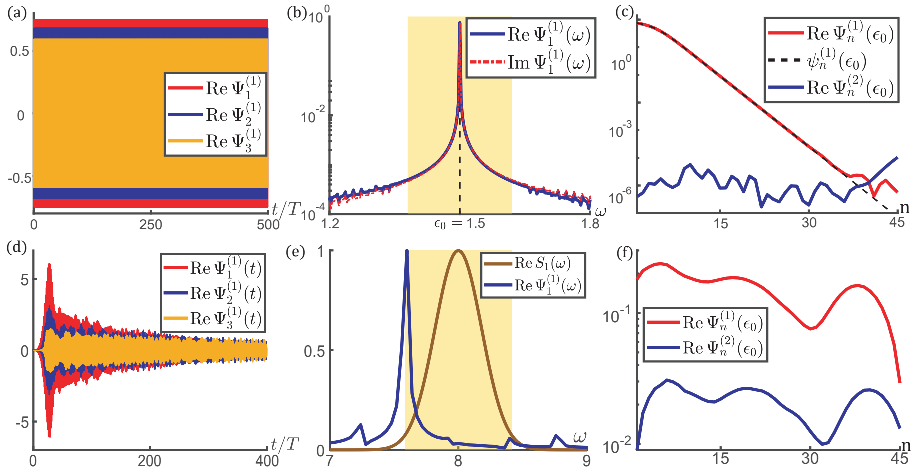

Supplementary Figure 8. Algorithm of self-oscillation that conducts the stability of nonlinear normal modes. (a-c) Stability analysis of nonlinear topological evanescent modes by performing the algorithm of self-oscillation in an undamped, undriven lattice. The lattice is constructed from $N = 45$ unit cells subjected to open boundary condition on both ends to mimic a semi-infinite lattice. The parameters of interactions are carried over from Fig.1 of the main text, namely $\epsilon_0 = 1.5$, $c_1 = 0.25$, $c_2 = 0.37$, $d_1 = 0.22$, and $d_2 = 0.02$. (a) A nonlinear topological edge mode with amplitude $\operatorname{Re}\Psi_1^{(1)} = 0.75 < A_c$. The mode is initialized by its analytic approximating form $\Psi_n \approx (\psi_{1,n}^{(1)}, 0)^\top e^{-i\epsilon_0 t}$ derived from Eq.(4.16), and is truncated in the finite lattice. The mode is allowed to self-oscillate in the lattice for more than $500T$, where $T = 2\pi/\epsilon_0$ is the theoretical prediction of the period. (b) Fourier analysis of the topological mode in frequency space, where the peak is in perfect agreement with $\omega_{\mathrm{T}} = \epsilon_0$, our theoretical anticipation of the mode frequency. The yellow shaded area is the linear band structure $|\epsilon_0 + c_1 - c_2| < \omega < |\epsilon_0 - c_1 + c_2|$. (c) Red and blue curves stand for the spatial profile of the peaks at $\omega = \epsilon_0$ of the Fourier components of the unit cells. Black dashed line is the analytic approximating solution $\Psi_n \approx (\psi_{1,n}^{(1)}, 0)^\top e^{-i\epsilon_0 t}$ derived from Eq.(4.16). (d-f) Nonlinear response of the open boundary of a semi-infinite lattice, where reflection symmetry is broken by replacing ϵ_0 with $\epsilon_A = (1 + 5\%)\epsilon_0$ on A-sites and $\epsilon_B = (1 - 5\%)\epsilon_0$ on B-sites, respectively. (d) The boundary manifest bulk-mode excitations in response to external Gaussian shaking signal. (e) Frequency spectrum of boundary response is composed of bulk mode components and is remarkably different from (b). (f) The spatial profile of the $\omega = \epsilon_0$ components is in strong contrast to (c).

We further truncate the equations of motion to the fundamental harmonics to find

$$\begin{aligned}
&(\epsilon_0 I + \omega_{\mathrm{T}}\sigma_y)\alpha_n + \\
&\begin{pmatrix} c_1(\sqrt{3}\beta_n^{(1)}/2)\beta_n^{(1)} + c_2(\sqrt{3}\beta_{n-1}^{(1)}/2)\beta_{n-1}^{(1)} \\ c_1(\sqrt{3}\beta_n^{(2)}/2)\beta_n^{(2)} + c_2(\sqrt{3}\beta_{n-1}^{(2)}/2)\beta_{n-1}^{(2)} \end{pmatrix} = 0, \\
&(\epsilon_0 I + \omega_{\mathrm{T}}\sigma_y)\beta_n + \\
&\begin{pmatrix} c_1(\sqrt{3}\alpha_n^{(1)}/2)\alpha_n^{(1)} + c_2(\sqrt{3}\alpha_{n+1}^{(1)}/2)\alpha_{n+1}^{(1)} \\ c_1(\sqrt{3}\alpha_n^{(2)}/2)\alpha_n^{(2)} + c_2(\sqrt{3}\alpha_{n+1}^{(2)}/2)\alpha_{n+1}^{(2)} \end{pmatrix} = 0, (4.14)
\end{aligned}$$

where $\beta_0 = 0$, and $c_i(x) = c_i + d_i|x|^2$, $i = 1, 2$. We solve Eqs.(4.14) by exploiting the approximation $\alpha_n \gg \beta_n$. By doing so, we obtain $\omega_{\rm T} = \epsilon_0$, $\alpha_n^{(1)} = i\alpha_n^{(2)}$, $\arg\alpha_n^{(1)} = \arg\alpha_1^{(1)} + (n-1)\pi$, and

$$c_1(\sqrt{3}\alpha_n^{(j)}/2)\alpha_n^{(j)} + c_2(\sqrt{3}\alpha_{n+1}^{(j)}/2)\alpha_{n+1}^{(j)} = 0 \quad (4.15)$$

for $j = 1, 2$, which in turn demands that

$$c_1(\sqrt{3}\psi_{1,n}^{(1)}/2)|\psi_{1,n}^{(1)}| = c_2(\sqrt{3}\psi_{1,n+1}^{(1)}/2)|\psi_{1,n+1}^{(1)}|, \quad (4.16)$$

where the parametrization in Eq.(4.12) has been used here.

Consequently, the analytic waveform of nonlinear topological edge mode is approximately solved as $\Psi_n \approx (\psi_{1,n}^{(1)}, 0)^\top e^{-i\epsilon_0 t}$. Let us denote $a_c = \sqrt{-(c_1-c_2)/(d_1-d_2)}$. If $|\psi_{1,1}^{(1)}| > \sqrt{4/3}a_c \approx A_c$, the mode keeps increasing and there is no topological edge mode, whereas for $|\psi_{1,1}^{(1)}| < \sqrt{4/3}a_c \approx A_c$, a topological evanescent mode fades away from the boundary. On the other hand, Berry phase of nonlinear bulk modes changes at the critical amplitude A_c. Above this critical amplitude, Berry phase $\gamma(A > A_c) = 0$. Below the transition point, Berry phase $\gamma(A < A_c) = \pi$. The relationship between the emergence of topological edge modes and Berry phase is the manifestation of the nonlinear extension of bulk-boundary correspondence.

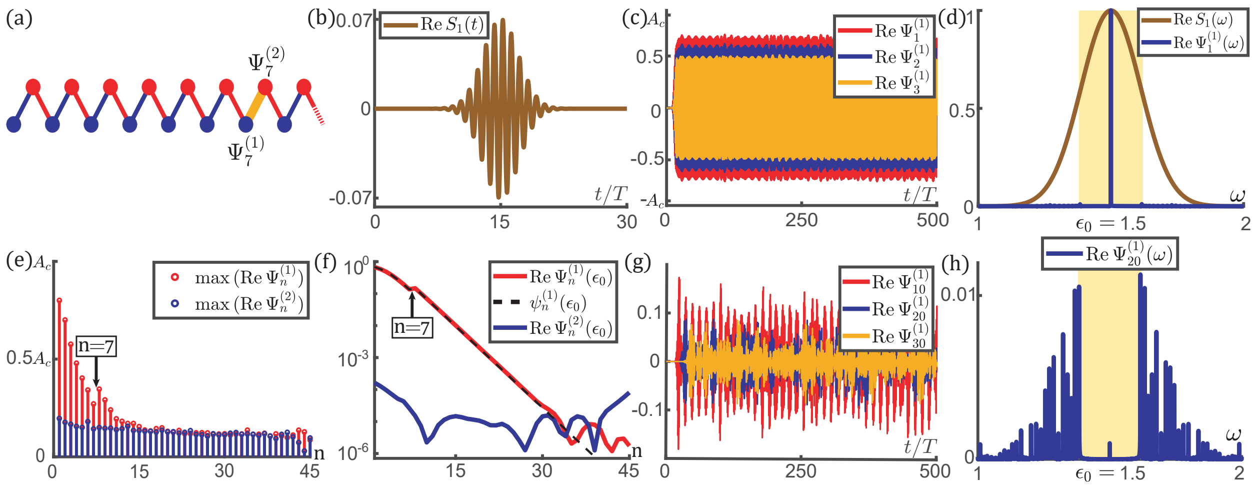

Supplementary Figure 9. Exciting topological edge modes in nonlinear SSH lattice with a defect. The interaction parameters $\epsilon_0, c_1, c_2, d_1$ and d_2 are the same as main text Fig.1. (a) We construct a long chain that consists of $N = 45$ unit cells and is subjected to the open boundary conditions on both ends to mimic a semi-infinite lattice. Red and blue bonds stand for nonlinear interactions between nearest neighbors. The defect is introduced by replacing the blue bond with an orange one connecting $\Psi_7^{(1)}$ and $\Psi_7^{(2)}$, where the interaction parameters are replaced by $c_1' = 0.4$ and $d_1' = 0.15$. (b) A Gaussian tone burst is employed on the first site to excite topological edge mode, where all parameters of this driving signal are carried over from main text Fig.2. (c) Wave functions of $n = 1, 2, 3$ sites exhibit the localization of topological mode, where the amplitude $\max(\mathrm{Re}\,\Psi_1^{(1)}) < A_c$. (d) Brown and blue curves represent the frequency profiles of Gaussian shaking and responding mode of site $n = 1$, respectively. Yellow shaded area is the linear bandgap. Despite the defect, the frequency of topological mode is still $\omega_{\rm T} = \epsilon_0 = 1.5$. (e) The spatial profile of mode amplitude captures a noticeable jump at site $n = 7$ which stems from the defect. (f) Red and blue curves are the spatial profiles of the $\omega = \epsilon_0$ wave component, where the noticeable jump is presented in the $\mathrm{Re}\,\Psi_n^{(1)}(\epsilon_0)$ curve at the 7th site. The analytic prediction of the topological mode $\psi_n^{(1)}(\epsilon_0)$ is described by the black dashed line, which is in perfect agreement with numerical results. (g) The wave functions of $n = 10, 20, 30$ sites exhibit echo-like shapes indicating multiple reflections at the boundaries, which in turn show the bulk mode excitations. These bulk mode components are excited by the input Gaussian tone burst in (b) which contains all frequencies. (h) The frequency spectrum indicates that the mode at site $n = 20$ is mainly composed of bulk modes.

We now present the numerical details of exciting nonlinear topological edge modes, given the parameters $\epsilon_0 = 1.5$, $c_1 = 0.25$, $c_2 = 0.37$, and $d_1 = 0.22$, $d_2 = 0.02$. We construct a lattice subjected to open boundary conditions on both ends. The lattice consists of $N = 45$ unit cells to mimic a semi-infinite lattice. According to our theory, the lattice is in the topological phase when the bulk wave amplitude $A < A_c \approx \sqrt{4/3}a_c$. Bulk-boundary correspondence demands that an evanescent mode should appear on lattice boundary, if the edge mode amplitude $\max(\mathrm{Re}\,\Psi_1^{(1)}) < A_c$. Theoretical analysis indicates that the spatial profile of this edge mode shall obey Eq.(4.16).

We now attempt to numerically verify this result by exciting a topological edge mode with amplitude $A < A_c$. To this end, a Gaussian tone burst

$$S_n = \delta_{n1} S e^{-i\omega_{\rm ext} t-(t-t_0)^2/\tau^2}(1,0)^\top \qquad (4.17)$$

is applied on the open boundary at site $n = 1$, where the driving amplitude $S = 7 \times 10^{-2}$, the carrier frequency $\omega_{\rm ext} = \epsilon_0$, the mode period $T = 2\pi/\omega_{\rm ext}$, the half height width $\tau = 3T$, and $t_0 = 15T$. In order to confirm the steady-state conditions, we wait $5000T$ before making any wave function measurements. We compute the frequency spectrum $\mathrm{Re}\,\Psi_1^{(1)}(\omega)$ by performing fast Fourier transformation (fft) for the time interval $t \in [10, 5000]T$ in main text Fig.2(d). In main text Fig.2(e), we plot the spatial profile of the amplitude of the boundary excitation, $\max(\mathrm{Re}\,\Psi_n^{(1,2)}(t))$. In main text Fig.2(f), we plot the spatial profile of the Fourier component $\mathrm{Re}\,\Psi_n^{(1,2)}(\omega = \epsilon_0)$. The curves are in perfect agreement with the theoretical predictions of nonlinear topological mode $\Psi_n \approx (\psi_{1,n}^{(1)}, 0)^\top e^{-i\epsilon_0 t}$, where $\psi_{1,n}^{(1)}$ are computed by Eq.(4.16).

The stability analysis of nonlinear topological edge modes is similar to what has been done in nonlinear bulk modes. We construct a lattice that is composed of $N = 45$ unit cells and is subjected to the open boundary conditions on both ends, to mimic a semi-infinite lattice. We initialize the topological mode by employing the analytic approximating solution $\Psi_n \approx (\psi_{1,n}^{(1)}, 0)^\top e^{-i\epsilon_0 t}$ derived from Eq.(4.16). After more than 10 periods of self-oscillation in the undamped, undriven lattice, we perform Fourier analysis to characterize if the mode has fallen apart to other nonlinear modes. As shown in Fig.8, the mode remains intact for more than $500T$, which demonstrates mode stability. What is more, all features of this nonlinear topological mode, including the frequency and the spatial profile of mode amplitude, are in perfect alignment with the approximated theoretical solution of Eq.(4.16).

According to our theory, nonlinear topological modes do not exist if $\max(\mathrm{Re}\,\Psi_1^{(1)}) > A_c$ in the T-to-N transition (topological-to-non-topological transition). We numerically verify this by driving the lattice boundary with a Gaussian tone burst (Eq.(4.17)), where the stimulation amplitude is $S = 53 \times 10^{-2}$. As shown in main text Figs.2(f), the amplitude of the responding signal is nearly the same for all sites, and the frequency spectrum comprises bulk modes. We note that due to the large amplitude of excitation, the responding nonlinear mode quickly shows instability [30] and falls apart to other nonlinear modes. To have a stable responding signal, we introduce small damping $\eta = 10^{-3}$ for this large-amplitude driven case. Damping is ubiquitous in dissipative classical systems (see Eqs.(5.7) for example).

In Figs.8(d-f), we study the nonlinear boundary response of the semi-infinite lattice, where reflection symmetry is broken by replacing the on-site potentials ϵ_0 with $\epsilon_A = (1 + \delta)\epsilon_0$ on A-sites and $\epsilon_B = (1 - \delta)\epsilon_0$ on B-sites, respectively. We drive the lattice with the same external Gaussian shaking presented in Eq.(4.17). Different from the reflection-symmetric models, the aforementioned symmetry-protected topological boundary modes quickly disappear due to the violation of reflection symmetry that quantizes Berry phase.

Fig.9 studies nonlinear topological edge modes in a lattice where the bond connecting $\Psi_7^{(1)}$ and $\Psi_7^{(2)}$ is replaced by the interaction $f_1'(\Psi_7^{(1)}, \Psi_7^{(2)}) = c_1' \Psi_7^{(2)} + d_1'[(\mathrm{Re}\,\Psi_7^{(2)})^3 + i(\mathrm{Im}\,\Psi_7^{(2)})^3]$. The topologically protected boundary mode is insensitive to the defect, in the sense that there is no change of frequency (i.e., $\omega_{\rm T} = \epsilon_0$), and the excitation is still robust.

C. Analytic solution of topological edge modes in the "purely nonlinear" model

An analytic solution of topological edge modes can be carried out when the linear components of interactions vanish, i.e., $c_1 = c_2 = 0$. The topological edge mode is expressed as $\Psi_n = (\psi_n^{(1)}, \psi_n^{(2)})^\top e^{-i\epsilon_0 t}$, where the mode amplitudes $\psi_n^{(1)}$ and $\psi_n^{(2)}$ satisfy the recursion relations,

$$\psi_{n+1}^{(1)}/\psi_n^{(1)} = \psi_n^{(2)}/\psi_{n+1}^{(2)} = (-d_1/d_2)^{1/3}\,. \qquad (4.18)$$

Given that $0 < d_1 < d_2$, the lattice is topologically nontrivial for all amplitudes because Berry phase always takes the non-trivial value, $\gamma(A) \equiv \pi$. As a result, an evanescent mode exponentially localizes on the open boundary, where the waveform is given by

$$\Psi_n = (\psi_1^{(1)}, 0)^\top (-d_1/d_2)^{(n-1)/3} e^{-i\epsilon_0 t}. \qquad (4.19)$$

In Figs.10(a–d), we study the nonlinear topological evanescent mode by driving an undamped lattice with a Gaussian shaking signal elaborated in Eq.(4.17). In Figs.10(e–h), we perform the algorithm of self-oscillation for the stability analysis. Both numerical methods suggest that the topological mode is stable in the "purely nonlinear regime" where the linear parts of hopping terms vanish.

D. Exact solution of static nonlinear topological edge modes for the $\epsilon_0 = 0$ case

In contrast to the $\epsilon_0 \neq 0$ model, this $\epsilon_0 = 0$ model features two qualitatively different properties.

The first property lies in the lattice under periodic boundary condition. At the critical amplitude a_c, the

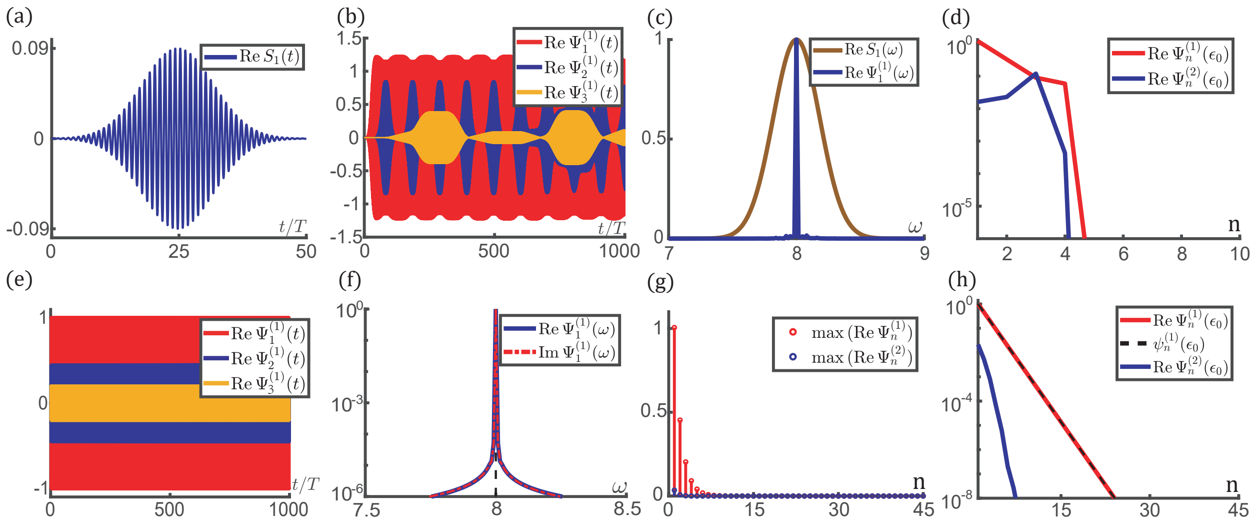

Supplementary Figure 10. Topological evanescent modes in the "purely nonlinear" model, where $\epsilon_0 = 8$, $c_1 = c_2 = 0$, $d_1 = 0.02$ and $d_2 = 0.22$. A chain composed of $N = 45$ unit cells is considered, where open boundary conditions are adopted on both ends. (a) A Gaussian shaking signal is applied on the $n = 1$ site to excite nonlinear topological modes, where $S = 9 \times 10^{-2}$, $\omega_{\text{ext}} = \epsilon_0 = 8$, $T = 2\pi/\omega_{\text{ext}}$, $\tau = 3T$, and $t_0 = 15T$. (b) Responding mode on $n = 1, 2, 3$ sites exhibits mode localization. (c) Brown and blue curves stand for the frequency spectra of external Gaussian signal and the responding wave function of the $n = 1$ site, respectively. The mode frequency is in in perfect alignment with theoretical predictions. (d) Red and blue curves are the spatial profiles of the $\omega = \epsilon_0$ Fourier component of the boundary mode, which manifest the evanescent nature of topological modes. (e) We execute the stability analysis by initializing the mode via Eq.(4.19) with $\psi_1^{(1)} = 1$, and impose an additional perturbation by multiplying a random factor $1 + \xi_n$ ($\xi_n \leq 10^{-2}$) on the wave function of each site n. We let the mode to self-oscillate in an undamped, undriven lattice for more than 1000 periods before measuring the wave functions. The mode remains intact without generating other components, which demonstrates the mode stability. (f) Frequency profile of the topological mode. (g) Spatial profile of the amplitude of this mode. (h) Spatial profile of the $\omega = \epsilon_0$ mode component is captured by red and blue curves. Theoretical analysis is depicted by the black dashed curve, which is in perfect agreement with numerical computations.

nonlinear bands merge at zero-frequency. When the mode amplitude goes beyond this critical amplitude, the lattice experiences instability to reach new ground states. There are eight new ground states described by the equilibrium wave functions,

$$\bar{\Psi}_n = (-1)^n \sqrt{2} a_c (e^{is_1\pi/4}, s_2 e^{is_3\pi/4})^\top, \tag{4.20}$$

where $s_1, s_2, s_3 = \pm 1$. Without loss of generality, we pick one of the eight equilibrium ground states, $\bar{\Psi}_n = (-1)^n e^{i\pi/4}\sqrt{2}a_c(1,1)^\top$, to study small fluctuations $\delta\Psi_n = \Psi_n - \bar{\Psi}_n$ around it. By expanding the equations to the linear order in $\delta\Psi_n$, we obtain

$$H_q \delta\Psi_q = i\partial_t \delta\Psi_q, \tag{4.21}$$

where $\delta\Psi_q = \sum_q \delta\Psi_n e^{-iqn}$ is the wavevector-space wave function, and the new ground state Hamiltonian H_q reads

$$H_q = [c_1(\sqrt{3}a_c) + c_2(\sqrt{3}a_c)\cos q]\sigma_x + [c_2(\sqrt{3}a_c)\sin q]\sigma_y. \tag{4.22}$$

The second peculiar property is that the nonlinear topological edge modes are static in time, which allows for analytic solutions governed by the following nonlinear recursion relations,

$$\begin{aligned}
c_1(\mathrm{Re}\,\Psi_n^{(1)})|\mathrm{Re}\,\Psi_n^{(1)}| &= c_2(\mathrm{Re}\,\Psi_{n+1}^{(1)})|\mathrm{Re}\,\Psi_{n+1}^{(1)}|, \\
c_1(\mathrm{Im}\,\Psi_n^{(1)})|\mathrm{Im}\,\Psi_n^{(1)}| &= c_2(\mathrm{Im}\,\Psi_{n+1}^{(1)})|\mathrm{Im}\,\Psi_{n+1}^{(1)}|, \\
c_1(\mathrm{Re}\,\Psi_n^{(2)})|\mathrm{Re}\,\Psi_n^{(2)}| &= c_2(\mathrm{Re}\,\Psi_{n-1}^{(2)})|\mathrm{Re}\,\Psi_{n-1}^{(2)}|, \\
c_1(\mathrm{Im}\,\Psi_n^{(2)})|\mathrm{Im}\,\Psi_n^{(2)}| &= c_2(\mathrm{Im}\,\Psi_{n-1}^{(2)})|\mathrm{Im}\,\Psi_{n-1}^{(2)}|,
\end{aligned} \tag{4.23}$$

subjected to the open boundary condition $\Psi_0^{(2)} = 0$. This is the nonlinear analog of chiral symmetric systems.

Stability analysis of topological modes is elaborated on as follows. We construct a lattice with $N = 45$ unit cells subjected to the open boundary conditions on both ends, and initialize the mode via the following procedure. We establish the analytic solution of Eqs.(4.23) with the amplitude $\mathrm{Re}\,\Psi_1^{(1)} = \mathrm{Im}\,\Psi_1^{(1)} = 0.99a_c$. Next, we perturb the aforementioned mode by multiplying a random factor $1 + \xi_n$ ($\xi_n \leq 10^{-2}$) on the wave function of each site n. Finally, we let the initialized mode to self-oscillate in the undamped, undriven lattice. We wait $t = 75 \times 2\pi/(c_2 - c_1)$ before making any wave function measurements. As shown in Fig.11, the mode remains intact without producing other wave components, which demonstrates the stability of nonlinear topological edge modes.

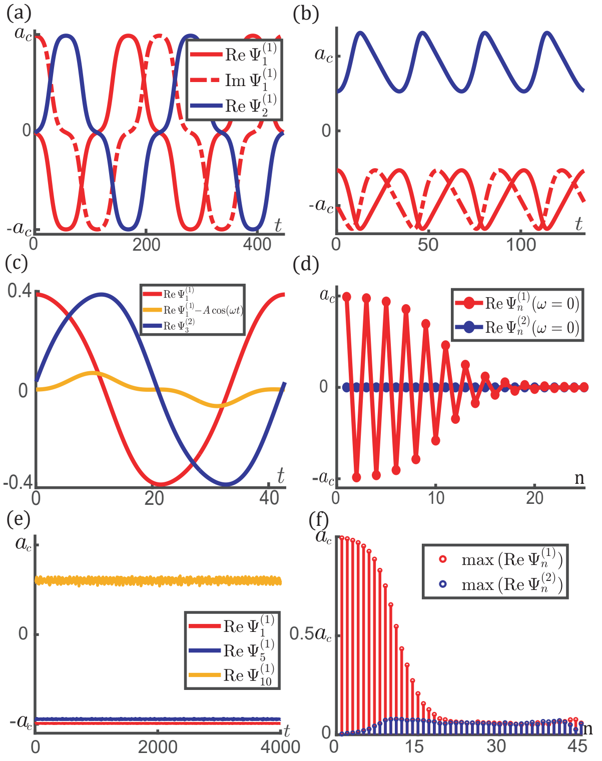

Supplementary Figure 11. We now consider the $\epsilon_0 = 0$ model, while all other parameters keep the same as main text Fig.1. (a) A nonlinear bulk mode on the verge of instability, where the amplitude $A = 0.9877a_c \lesssim a_c$. (b) A nonlinear bulk mode with $\max|\mathrm{Re}\,\Psi_n^{(1)}| = 1.312a_c > a_c$ experiences instability and oscillates around new ground states. (c) A nonlinear bulk mode with $A = 0.5a_c$ and $q = 8\pi/9$ is obtained via shooting method. Red and blue curves stand for the wave functions of $n = 1, 3$ sites. Orange curve indicates that the nonlinear mode is noticeably different from sinusoidal function. (d) Spatial profile of static topological mode with amplitude $\mathrm{Re}\,\Psi_1^{(1)} = 0.99a_c$. (e) Stability analysis of nonlinear topological edge modes. Temporal profile of the perturbed topological mode for the time interval $t \in [0, 75 \times 2\pi/(c_2 - c_1)]$. The mode remains intact without generating other wave components, which demonstrates mode stability. (f) Spatial profile of the amplitude of the mode in (c) on each site.

E. Definition of nonlinear topological boundary modes

Here, we define topological boundary modes in strongly nonlinear systems. To this end, we summarize the properties of linear and nonlinear topological boundary states that bulk modes do not have. (1) Linear and nonlinear topological edge states cannot arise in uniform systems with periodic boundary conditions. (2) They emerge on lattice boundaries only when the topological number is quantized to the non-trivial integer value, while these modes never exist for trivially quantized or un-quantized Berry phases. (3) In contrast to bulk modes that manifest spatially uniform amplitudes, the mode amplitudes are non-uniform for topological boundary modes. (4) The frequencies of linear and nonlinear topological modes stay in the band gap and therefore are not mixed with bulk states. (5) Linear and nonlinear topological modes are robust against perturbations as long as the band gap is not closed.

In summary, we define nonlinear topological edge states as temporal-periodic nonlinear modes that satisfy the above five properties.

V. DERIVING GENERALIZED NONLINEAR SCHRÖDINGER EQUATIONS FOR CLASSICAL MODELS

In this section, we take a two-step derivation to show that the nonlinear motion equations for the topoelectrical circuit can be written as generalized nonlinear Schrödinger equations. First, we discuss a purely linear topoelectrical circuit that hosts quantized Berry phase. Second, we add nonlinear voltage sources to the linear system and realize nonlinear topological attributes.

In the first part, we start with Fig.12(a) to consider a purely linear topoelectrical circuit. The unit cell is composed of two LCR resonators with the natural frequency $\omega_0 = 1/\sqrt{LC}$, where L and C are the inductance and capacitance, respectively. These resonators are connected by linear capacitors C_1 and C_2 that yield the condition $C_{j=1,2} \ll C$. We derive the motion equations for $V_n^{(1)}$ and $V_n^{(2)}$ marked in Fig.12(a), which are the voltage fields of the resonators.

We denote the currents in the inductances as $i_n^{(j)}$, and the currents of the capacitors as $I_n^{(j)}$ for $j = 1, 2$. We further denote $V_n'^{(1)}$, $I_n'^{(1)}$ and $V_n'^{(2)}$, $I_n'^{(2)}$ as the voltages and currents of the capacitors C_1 and C_2, respectively. Kirchhoff's law demands that the in-flow and out-flow currents conserve on the junctions:

$$\begin{aligned} I_n'^{(1)} &= i_n^{(1)} + I_n^{(1)} + I_n'^{(2)}, \\ I_n'^{(2)} &= i_n^{(2)} + I_n^{(2)} + I_{n+1}'^{(1)}. \end{aligned} \tag{5.1}$$

The voltage fields yield

$$\begin{aligned} V_n'^{(1)} &= V_{n-1}^{(2)} - V_n^{(1)}, \\ V_n'^{(2)} &= V_n^{(1)} - V_n^{(2)}. \end{aligned} \tag{5.2}$$

The charges of the capacitors are proportional to their voltages, $Q_n^{(j)} = CV_n^{(j)}$ and $Q_n'^{(j)} = CV_n'^{(j)}$. Thus, the currents in the capacitors are given by

$$\begin{aligned} I_n^{(j)} &= C\dot{V}_n^{(j)}, \\ I_n'^{(j)} &= C_j\dot{V}_n'^{(j)}, \end{aligned} \tag{5.3}$$

for $j = 1, 2$. Substituting Eqs.(5.1, 5.2, 5.3) into the motion equations of the inductances,

$$(L\partial_t + R)i_n^{(j)} = V_n^{(j)}, \qquad j = 1, 2, \tag{5.4}$$

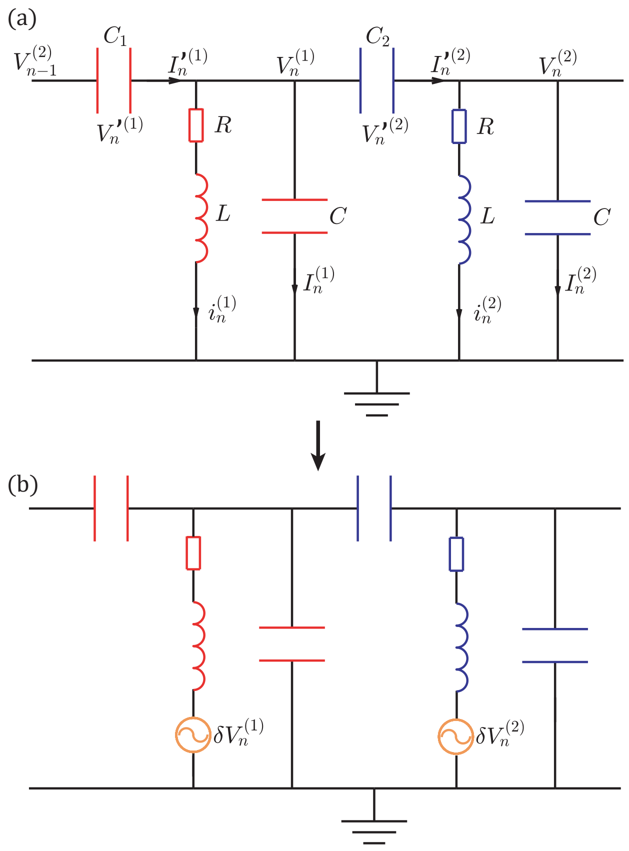

Supplementary Figure 12. Experimental proposals of topological electrical circuits. (a) Unit cell of purely linear topoelectrical circuit without nonlinear elements. (b) The unit cell of nonlinear topoelectrical circuit is constructed by introducing active voltage sources $\delta V_n^{(1)}(V_n^{(2)}, V_{n-1}^{(2)})$ and $\delta V_n^{(2)}(V_n^{(1)}, V_{n+1}^{(1)})$, which are nonlinearly controlled by voltage fields $V_n^{(j=1,2)}$.

we obtain the motion equations of the ladder circuit:

$$(L\partial_t^2 + R\partial_t)(2C_0\omega_0^{-1}H - C_0 I)\mathbf{V} = \mathbf{V}, \qquad (5.5)$$

where $C_0 = C + C_1 + C_2$, $\mathbf{V} = (\dots, V_n^{(1)}, V_n^{(2)} \dots)^\top$ is the state vector of the voltage fields, and

$$H = \frac{\omega_0}{2C_0}\begin{pmatrix} \dots & \dots & \dots & \dots & \dots & \dots & \dots \\ \dots & 0 & C_2 & 0 & 0 & 0 & \dots \\ \dots & C_2 & 0 & C_1 & 0 & 0 & \dots \\ \dots & 0 & C_1 & 0 & C_2 & 0 & \dots \\ \dots & 0 & 0 & C_2 & 0 & C_1 & \dots \\ \dots & 0 & 0 & 0 & C_1 & 0 & \dots \\ \dots & \dots & \dots & \dots & \dots & \dots & \dots \end{pmatrix}. \qquad (5.6)$$

The reflection-symmetric property of H is easily demonstrated by converting it to wavevector space $H(q) = \omega_0[(C_1\cos q + C_2)\sigma_x + (C_1 \sin q)\sigma_y]/2C_0$ and showing that $\sigma_x H(q)\sigma_x^{-1} = H(-q)$.

Up to now, we have not employed any approximation. Based on Ref. [1], we use the "small-capacity condition" $C_{j=1,2} \ll C$ by dropping terms proportional to C_1^2/C_0^2, C_2^2/C_0^2 and C_1C_2/C_0^2, to reduce Eqs.(5.5) as $-(L_0C_0^2\partial_t^2 + RC_0^2\partial_t + C_0)\mathbf{V} = 2C_0\omega_0^{-1}H\mathbf{V}$. We then express the voltage fields as the envelope functions $\mathbf{V} = \Psi e^{-i\omega_0 t}$. We assume that the time-modulation of $\mathbf{V}$ is mostly captured by the factor $e^{-i\omega_0 t}$ and then Ψ varies slowly in time to yield $\ddot{\Psi} \ll \omega_0\dot{\Psi}$. Finally, we consider the undamped limit $RC\omega_0 \to 0$ to express the circuit dynamics as the linear Schrödinger equation,

$$i\partial_t\Psi = H\Psi. \qquad (5.7)$$

Clearly, linear topological voltage fields arise on lattice open boundary due to the topological nature in H.

In the second part, we introduce nonlinear elements into the circuit model to have nonlinear topological attributes. As pictorially depicted in Fig.12(b), we add external alternating voltage sources $\delta V_n^{(1)}$ and $\delta V_n^{(2)}$ that are nonlinearly controlled by voltage fields $V_n^{(j=1,2)}$. Without these external voltage sources (see Fig.12(a)), the purely linear circuit manifests topological boundary voltage excitations due to the reflection-symmetric nature of the lattice dynamics. Thus, it is intuitive to expect that topological boundary voltages also emerge if the nonlinear voltage sources $\delta V_n^{(1)}$ and $\delta V_n^{(2)}$ respect reflection symmetry. We realize this symmetry condition by asking $\delta V_n^{(1)} = \delta V_n^{(1)}(V_n^{(2)}, V_{n-1}^{(2)})$ and $\delta V_n^{(2)} = \delta V_n^{(2)}(V_n^{(1)}, V_{n+1}^{(1)})$, but their functional forms can be arbitrary. These active power sources provide nonlinear voltage feedback to the motion equations. Consequently, the right hand side of Eqs.(5.4) is modified from $V_n^{(j)}$ to $V_n^{(j)} - \delta V_n^{(j)}$, for $j = 1, 2$. Likewise, the right hand side of Eq.(5.5) changes from $\mathbf{V}$ to $\mathbf{V} - \delta\mathbf{V}$, where $\delta\mathbf{V} = (\delta V_1^{(1)}, \delta V_1^{(2)}, \dots, \delta V_n^{(1)}, \delta V_n^{(2)}, \dots)^\top$ is the state vector of the nonlinear voltage fields. We repeat the small-capacity approximation by dropping terms in C_1^2/C^2, C_2^2/C^2, and C_1C_2/C^2, and use envelope function representations for $\mathbf{V} = \Psi e^{-i\omega_0 t}$ and $\delta\mathbf{V} = -\delta\Psi e^{-i\omega_0 t}$. In the undamped limit, we obtain the generalized nonlinear Schrödinger equation

$$i\partial_t\Psi = H\Psi + \delta\Psi, \qquad (5.8)$$

where $\delta\Psi$ is nonlinearly controlled by the field variable Ψ to offer nonlinear interactions. $\delta\Psi$ respects reflection symmetry, nonlinear Berry phase is quantized, and topological voltage fields are manifested on the lattice open boundary. In particular, the nonlinear interaction presented in Eq.(7) of the main text is realized by asking

$$\begin{aligned} \delta\Psi_{2n-1} &= f_1(\Psi_{2n-1}, \Psi_{2n}) + f_2(\Psi_{2n-1}, \Psi_{2n-2}), \\ \delta\Psi_{2n} &= f_1(\Psi_{2n}, \Psi_{2n-1}) + f_2(\Psi_{2n}, \Psi_{2n+1}). \end{aligned} \qquad (5.9)$$

Eqs.(5.9) specify the relationships between $\delta V_n^{(j)}$ and $V_n^{(j)}$, and demand active electrical elements.

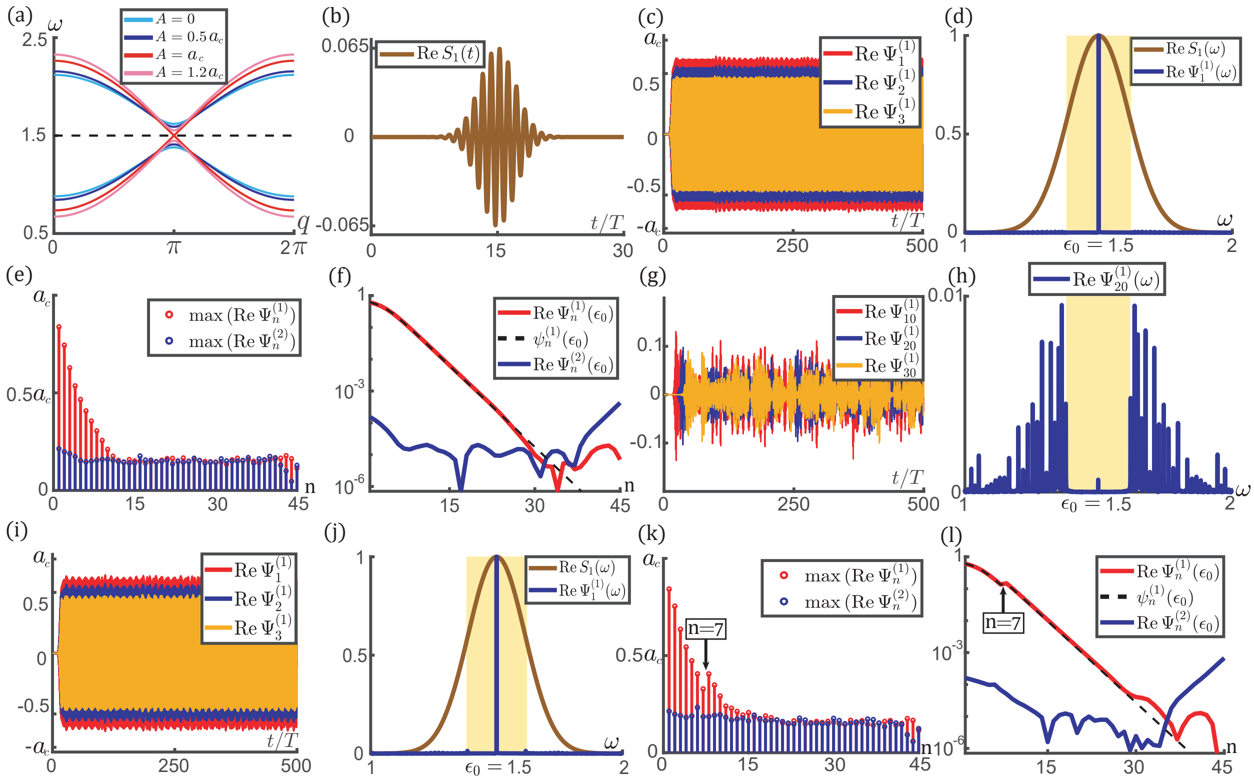

Supplementary Figure 13. Topological properties of nonlinear SSH lattice subjected to Kerr-type nonlinearities. The interaction parameters, including ϵ_0, c_1, c_2, d_1 and d_2, are the same as those in main text Fig.1. (a) Nonlinear band structures for various amplitudes ranging from $A = 0$ to $1.2a_c$, where $a_c = \sqrt{-(c_2 - c_1)/(d_2 - d_1)} = 0.7746$. The nonlinear bands touch at the critical amplitude $A = a_c$, and Berry phase changes abruptly from $\gamma(A < a_c) = \pi$ to $\gamma(A > a_c) = 0$. (b) By constructing a finite lattice shown in main text Fig.1(a) with open boundary conditions, we shake the boundary on site $n = 1$ by imposing a Gaussian tone burst in Eq.(4.17) to excite nonlinear topological modes, where $S = 6.5 \times 10^{-2}$, $\omega_{\text{ext}} = \epsilon_0 = 1.5$, $T = 2\pi/\omega_{\text{ext}}$, $\tau = 3T$, and $t_0 = 15T$. (c) Responding mode on $n = 1, 2, 3$ sites indicates the mode localization, where the mode amplitude $\max(\text{Re}\,\Psi_1^{(1)}) < a_c$. (d) Brown and blue curves stand for the frequency spectra of external Gaussian signal and $n = 1$ site responding wave function. Yellow shaded area is the linear bandgap. (e) Spatial profile of the boundary excitation amplitude. We note that the bulk mode components reflected here are excited by Gaussian signal, which contains all frequencies. (f) Red and blue curves are the spatial profiles of the $\omega = \epsilon_0$ Fourier component of the boundary mode. The analytic result of the $\omega = \epsilon_0$ Fourier component is depicted by the black dashed curve. (g) The echo-like wave functions of sites $n = 10, 20, 30$ manifest bulk mode components excited by the external Gaussian shaking signal. (h) The spectrum of $n = 20$ site contains a wide range of frequency components of bulk modes. (i) We now study topological edge modes in the lattice depicted by Fig.9(a), where the interaction parameters c_1' and d_1' of the defect bond at site $n = 7$ are carried over from that figure. Responding modes are plotted for $n = 1, 2, 3$ sites which exhibit the feature of mode localization. (j) Fourier analysis of frequency space for wave function at site $n = 1$. (k) Spatial profile of the responding amplitudes. A noticeable bump at site $n = 7$ is induced by the defect. (l) Spatial profile of the $\omega = \epsilon_0$ frequency component is captured by red and blue curves, and the theoretical analysis of this component is described by the black dashed curve.

VI. TOPOLOGICAL INVARIANT AND NONLINEAR EDGE MODES IN MODELS OF KERR-TYPE NONLINEAR INTERACTIONS

We now demonstrate that our universal theory not only studies nonlinear systems beyond Kerr-like interactions, but also covers existing results of Kerr-nonlinearities in topological photonics. The model is analytically solvable, in the sense that the nonlinear bulk modes as well as the dispersion relation can be exactly solved. The model is based on Eqs.(1.1) with the Kerr-type nonlinearities [31] on the field variables,

$$f_i(x, y) = c_i y + d_i |y|^2 y, \quad i = 1, 2, \tag{6.1}$$

where the parameters yield $0 < c_1 < c_2$ and $d_1 > d_2 > 0$. This model is subjected to reflection symmetry. According to the main text, reflection symmetry demands the quantization of Berry phase of nonlinear bulk modes, regardless of the functional forms of interactions. This conclusion should remain valid for Kerr-type nonlinearities. To verify the quantization of Berry phase, we solve non-

linear traveling modes as below,

$$\Psi_n = A(1, e^{-i\phi_q})^\top e^{iqn-i\omega t}, \tag{6.2}$$

where the dispersion relation is

$$\omega = \epsilon_0 \pm \sqrt{c_1(A)^2 + c_2(A)^2 + 2c_1(A)c_2(A)\cos q}, \tag{6.3}$$

$c_i(A) = c_i + d_i A^2$, and the relative phase ϕ_q is

$$\phi_q = \arctan\left(\frac{-c_2(A)\sin q}{c_1(A) + c_2(A)\cos q}\right). \tag{6.4}$$

Following the convention of Fourier transformation in Eq.(1.8), the Fourier components of the sinusoidal nonlinear bulk mode are $\psi^{(1)}_{l,q} = \psi^{(2)}_{l,q} = A\delta_{l,1}$. According to Eq.(6.3), the nonlinear bandgap never closes unless the wave amplitude hits the topological transition point a_c. Apart from a_c, Berry phase of nonlinear bulk modes is well-defined, and can be greatly simplified to the following result by employing the sinusoidal form of nonlinear waves,

$$\begin{aligned}\gamma(A) &= \frac{1}{2}i\oint_{\text{BZ}} dq\,\partial_q \ln[c_1(A) + c_2(A)e^{iq}] \\ &= \frac{1}{2}i\oint_{\text{BZ}} dq\,\partial_q \ln\det C(q, A),\end{aligned} \tag{6.5}$$

where $C(q, A) = c_1(A) + c_2(A)e^{iq}$, and Eq.(6.5) appears as the standard format of the winding number of the ordinary Kerr-nonlinear SSH model.

According to our general theory, Berry phase is expected to be $\gamma(A < a_c) = \pi$ and $\gamma(A > a_c) = 0$, which holds true for arbitrary reflection-symmetric 1D systems and is independent of the functional forms of nonlinearities. This result is verified by evaluating Eq.(6.5) for Kerr-type nonlinear interactions.

As stated by the nonlinear extension of bulk-boundary correspondence, nonlinear topological modes should emerge on the lattice open boundary when the bulk band is topologically non-trivial with Berry phase $\gamma = \pi$, whereas topological modes disappear when $\gamma = 0$. Here we confirm this correspondence by studying the attributes of nonlinear topological edge modes. To this end, we Fourier transform the edge mode into frequency space, and truncating it to the fundamental harmonics,

$$\Psi_n \approx \psi_{-1,n}e^{i\omega t} + \psi_{1,n}e^{-i\omega t}. \tag{6.6}$$

Similarly, the nonlinear terms in the interactions are truncated as follows,

$$\begin{aligned}|\Psi^{(j)}_n|^2\Psi^{(j)}_n \approx\ & (|\psi^{(j)}_{-1,n}|^2 + 2|\psi^{(j)}_{1,n}|^2)\psi^{(j)}_{-1,n}e^{i\omega t} \\ &+ (2|\psi^{(j)}_{-1,n}|^2 + |\psi^{(j)}_{1,n}|^2)\psi^{(j)}_{1,n}e^{-i\omega t}.\end{aligned} \tag{6.7}$$

Consequently, the equations of motion reduce to the following nonlinear recursion relations,

$$\begin{aligned}(\epsilon_0 - s\omega)\psi^{(1)}_{s,n} + C_1(\psi^{(2)}_{s,n}) + C_2(\psi^{(2)}_{s,n-1}) = 0, \\ (\epsilon_0 - s\omega)\psi^{(2)}_{s,n} + C_1(\psi^{(1)}_{s,n}) + C_2(\psi^{(1)}_{s,n+1}) = 0,\end{aligned} \tag{6.8}$$

where $s = \pm 1$, and

$$C_i(\psi^{(j)}_{s,n}) = c_i\psi^{(j)}_{s,n} + d_i(|\psi^{(j)}_{s,n}|^2 + 2|\psi^{(j)}_{-s,n}|^2)\psi^{(j)}_{s,n}. \tag{6.9}$$

We exploit the approximation $\psi^{(1)}_{s,n} \gg \psi^{(2)}_{s,n}$, which is numerically verified in Fig.13(f). We solve Eqs.(6.8) to find $\omega = \epsilon_0$, $\psi^{(1)}_{-1,n} = 0$ for all n, $\text{Arg}\,\psi^{(1)}_{1,n} = \text{Arg}\,\psi^{(1)}_{1,1} + (n-1)\pi$, and

$$c_1(\psi^{(1)}_{1,n})|\psi^{(1)}_{1,n}| = c_2(\psi^{(1)}_{1,n+1})|\psi^{(1)}_{1,n+1}|, \tag{6.10}$$

where $c_i(x) = c_i + d_i|x|^2$. Based on Eq.(6.10), when $|\psi^{(1)}_{1,1}| < a_c$, an evanescent mode fades away from the lattice boundary, whereas for $|\psi^{(1)}_{1,1}| > a_c$, an unphysical mode quickly diverges to infinity and therefore cannot exist. The emergence and disappearance of edge modes are in accordance with topologically non-trivial and trivial Berry phases, which is the manifestation of bulk-boundary correspondence with Kerr-type nonlinearities.

SUPPLEMENTARY REFERENCES

[1] Hadad, Y., Soric, J. C., Khanikaev, A. B., and Alù, A. Self-induced topological protection in nonlinear circuit arrays. *Nature Electronics* **1**, 3, 178–182 (2018).

[2] Hadad, Y., Khanikaev, A. B., and Alù, A. Self-induced topological transitions and edge states supported by nonlinear staggered potentials. *Phys. Rev. B* **93**, 155112 (2016).

[3] Frasca, M. Exact solutions of classical scalar field equations. *Journal of Nonlinear Mathematical Physics* **18**, 02, 291–297 (2011).

[4] Tsapalis, A., Politis, E., Maintas, X., and Diakonos, F. Gauss' law and nonlinear plane waves for yang-mills theory. *Physical Review D* **93**, 8, 085003 (2016).

[5] Schlutow, M., and Wahlén, E. Generalized modulation theory for strongly nonlinear gravity waves in a compressible atmosphere. *Mathematics of Climate and Weather Forecasting* **6**, 1, 97–112 (2020).

[6] Berjamin, H. Nonlinear plane waves in saturated porous media with incompressible constituents. *Proceedings of the Royal Society A* **477**, 20210086 (2021).

[7] Narisetti, R. K., Leamy, M. J., and Ruzzene, M. A perturbation approach for predicting wave propagation in one-dimensional nonlinear periodic structures. *Journal of Vibration and Acoustics* **132**, 3 (2010).

[8] Fronk, M. D., and Leamy, M. J. Higher-order dispersion, stability, and waveform invariance in nonlinear monoatomic and diatomic systems. *Journal of Vibration and Acoustics* **139**, 5 (2017).

[9] Vila, J., Paulino, G. H., and Ruzzene, M. Role of nonlinearities in topological protection: Testing magnetically

coupled fidget spinners. *Phys. Rev. B* **99**, 125116 (2019).

[10] Pal, R. K., Vila, J., Leamy, M., and Ruzzene, M. Amplitude-dependent topological edge states in nonlinear phononic lattices. *Phys. Rev. E* **97**, 032209 (2018).

[11] Zhou, D., Ma, J., Sun, K., Gonella, S., and Mao, X. Switchable phonon diodes using nonlinear topological maxwell lattices. *Phys. Rev. B* **101**, 104106 (2020).

[12] Zaera, R., Vila, J., Fernandez-Saez, J., and Ruzzene, M. Propagation of solitons in a two-dimensional nonlinear square lattice. *International Journal of Non-Linear Mechanics* **106**, 188–204 (2018).

[13] Vakakis, A. F., Manevitch, L. I., Mikhlin, Y. V., Pilipchuk, V. N., and Zevin, A. A. *Normal modes and localization in nonlinear systems.* Springer (2001).

[14] Renson, L., Kerschen, G., and Cochelin, B. Numerical computation of nonlinear normal modes in mechanical engineering. *Journal of Sound and Vibration* **364**, 177–206 (2016).

[15] Ha, S. N. A nonlinear shooting method for two-point boundary value problems. *Computers & Mathematics with Applications* **42**, 10-11, 1411–1420 (2001).

[16] Peeters, M., Viguié, R., Sérandour, G., Kerschen, G., and Golinval, J.-C. Nonlinear normal modes, part ii: Practical computation using numerical continuation techniques. In *26th International Modal Analysis Conference, Orlando, 2008* (2008).

[17] Xiao, D., Chang, M.-C., and Niu, Q. Berry phase effects on electronic properties. *Rev. Mod. Phys.* **82**, 1959–2007 (2010).

[18] Liu, J., Wu, B., and Niu, Q. Nonlinear evolution of quantum states in the adiabatic regime. *Phys. Rev. Lett.* **90**, 170404 (2003).

[19] Pu, H., Maenner, P., Zhang, W., and Ling, H. Y. Adiabatic condition for nonlinear systems. *Phys. Rev. Lett.* **98**, 050406 (2007).

[20] Liu, J., and Fu, L. B. Berry phase in nonlinear systems. *Phys. Rev. A* **81**, 052112 (2010).

[21] Maczewsky, L. J., Heinrich, M., Kremer, M., Ivanov, S. K., Ehrhardt, M., Martinez, F., Kartashov, Y. V., Konotop, V. V., Torner, L., Bauer, D., et al. Nonlinearity-induced photonic topological insulator. *Science* **370**, 6517, 701–704 (2020).

[22] Kane, C., and Lubensky, T. Topological boundary modes in isostatic lattices. *Nature Physics* **10**, 1, 39 (2014).

[23] Hasan, M. Z., and Kane, C. L. Colloquium: Topological insulators. *Rev. Mod. Phys.* **82**, 3045–3067 (2010).

[24] Lazarus, A., and Thomas, O. A harmonic-based method for computing the stability of periodic solutions of dynamical systems. *Comptes Rendus Mécanique* **338**, 9, 510–517 (2010).

[25] Kawai, R., Lindenberg, K., and Van den Broeck, C. Parametrically modulated oscillator dimer: an analytic solution. *Physica A: Statistical Mechanics and its Applications* **312**, 1-2, 119–140 (2002).

[26] Ryu, S., Schnyder, A. P., Furusaki, A., and Ludwig, A. W. Topological insulators and superconductors: tenfold way and dimensional hierarchy. *New Journal of Physics* **12**, 6, 065010 (2010).

[27] Snee, D. D., and Ma, Y.-P. Edge solitons in a nonlinear mechanical topological insulator. *Extreme Mechanics Letters* **30**, 100487 (2019).

[28] Luther, H. An explicit sixth-order runge-kutta formula. *Mathematics of Computation* **22**, 102, 434–436 (1968).

[29] Detroux, T., Renson, L., and Kerschen, G. The harmonic balance method for advanced analysis and design of nonlinear mechanical systems. In *Nonlinear Dynamics, Volume 2.* Springer, pp. 19–34 (2014).

[30] Crawford, J. D. Introduction to bifurcation theory. *Rev. Mod. Phys.* **63**, 991–1037 (1991).

[31] D'Aguanno, G., Mattiucci, N., and Bloemer, M. J. Ultraslow light pulses in a nonlinear metamaterial. *JOSA B* **25**, 8, 1236–1241 (2008).
